# CD8^+^ T cells specific for the islet autoantigen IGRP are restricted in their T cell receptor chain usage

**DOI:** 10.1038/srep44661

**Published:** 2017-03-16

**Authors:** Yannick F. Fuchs, Anne Eugster, Sevina Dietz, Christian Sebelefsky, Denise Kühn, Carmen Wilhelm, Annett Lindner, Anita Gavrisan, Jan Knoop, Andreas Dahl, Anette-G. Ziegler, Ezio Bonifacio

**Affiliations:** 1 DFG Center for Regenerative Therapies Dresden, Faculty of Medicine, Technische Universität Dresden, Dresden, Germany; 2 Paul Langerhans Institute Dresden of the Helmholtz Center Munich at University Hospital and Faculty of Medicine, Technische Universität Dresden, Dresden, Germany; 3 German Center for Diabetes Research (DZD e.V.), Neuherberg, Germany; 4 Institute of Diabetes Research, Helmholtz Zentrum München, Neuherberg, Germany; 5 Forschergruppe Diabetes e.V., Neuherberg, Germany; 6 Deep-Sequencing Facility SFB655, Biotechnology Center, Technische Universität Dresden, Dresden, Germany; 7 Klinikum rechts der Isar, Technische Universität München, Munich, Germany

## Abstract

CD8^+^ T cells directed against beta cell autoantigens are considered relevant for the pathogenesis of type 1 diabetes. Using single cell T cell receptor sequencing of CD8^+^ T cells specific for the IGRP_265-273_ epitope, we examined whether there was expansion of clonotypes and sharing of T cell receptor chains in autoreactive CD8^+^ T cell repertoires. HLA-A*0201 positive type 1 diabetes patients (n = 19) and controls (n = 18) were analysed. TCR α- and β-chain sequences of 418 patient-derived IGRP_265-273_-multimer^+^ CD8^+^ T cells representing 48 clonotypes were obtained. Expanded populations of IGRP_265-273_-specific CD8^+^ T cells with dominant clonotypes that had TCR α-chains shared across patients were observed. The SGGSNYKLTF motif corresponding to *TRAJ53* was contained in 384 (91.9%) cells, and in 20 (41.7%) patient-derived clonotypes. *TRAJ53* together with *TRAV29/DV5* was found in 15 (31.3%) clonotypes. Using next generation TCR α-chain sequencing, we found enrichment of one of these TCR α-chains in the memory CD8^+^ T cells of patients as compared to healthy controls. CD8^+^ T cell clones bearing the enriched motifs mediated antigen-specific target cell lysis. We provide the first evidence for restriction of T cell receptor motifs in the alpha chain of human CD8^+^ T cells with specificity to a beta cell antigen.

Autoreactive CD8^+^ T cells are in all likelihood key mediators of the pancreatic beta cell destruction leading to type 1 diabetes^1^,^2^,^3^,^4^,^5^,^6^. T cell receptor (TCR)-mediated recognition of (auto-) antigenic peptides presented on MHC class I molecules is a prerequisite for CD8^+^ T cell mediated target cell destruction. Several islet autoantigen epitopes presented on MHC class I molecules^5^,^7^,^8^,^9^,^10^,^11^,^12^,^13^ and assays to measure and quantify CD8^+^ T cell responses against these epitopes have been described^14^,^15^,^16^,^17^. However, information on the TCR repertoire of autoantigen specific CD8^+^ T cells in type 1 diabetes is so far limited to the TCR sequencing of propagated CD8^+^ T cell clones^18^, TCR sequence information of single TCR chains of isolated bulk autoantigen specific CD8^+^ T cells^19^, or CDR3 spectrotype data on bulk autoantigen specific CD8^+^ T cells^20^. These studies do not provide clonotype information and have not been able to show restricted TCR usage by the autoreactive CD8^+^ T cells.

TCRs are heterodimers consisting of TCR α- and β-chains and TCR diversity results from combinatorial rearrangements of variable (V), joining (J), and, for TCR β, also the diversity (D) gene segments. V-(D)-J sequences of both chains constitute the hypervariable complementary determining region 3 (CDR3) which provides the major contact point with the antigenic peptide and, therefore, determines antigen specificity of the T cell. The unique combination defines a clonotype. Although TCR clonotypes can be promiscuous in their binding to MHC-peptide complexes^21^, TCRs that recognize epitopes of viral and tumour antigens often have preferred CDR3 motifs or gene usage^22^,^23^,^24^,^25^,^26^, indicating that some structural restriction of the MHC-peptide binding region of the TCR plays an important role in the selection and expansion of clones.

In this study, we interrogated the TCR repertoire of CD8^+^ T cells directed against an epitope of an islet autoantigen using single cell TCR sequencing in order to determine whether there is TCR selection in islet autoantigen-specific CD8^+^ T cells. We chose the islet-specific glucose-6-phosphatase catalytic subunit related protein (IGRP) antigen as a model islet autoantigen, since an HLA A*0201 restricted peptide, IGRP_265-273_, has been identified and IGRP_265-273_ directed CD8^+^ T cells have been detected in the pancreatic islets of organ donors with type 1 diabetes^27^. Additionally, the occurrence and quantification of CD8^+^ T cells directed against the islet autoantigen IGRP has been demonstrated to have prognostic value on autoimmune diabetes development in NOD mice^4^,^28^. Our findings suggest that, as described for virus-specific CD8^+^ T cells, there is selection and expansion of a restricted TCR repertoire in islet-antigen specific CD8^+^ T cells.

## Results

### T cell receptor sequencing reveals dominant clonotypes and common alpha chains for IGRP-specific CD8^+^ T cells

We initially tested our TCR sequencing approach using CD8^+^ T cells that stained positive with MHC class I multimers loaded with a *bona fide* Influenza peptide epitope (Flu MP_58-66_; Supplementary Fig. S1a). From the analysed cells, we identified new as well as previously described^22^,^23^,^29^,^30^ Flu MP_58-66_-specific T cell receptor chains (see Supplementary Table S1). We noted inter-individual sharing of TCR α-chains among the analysed Flu-specific cells (Supplementary Fig. S1b) and, in accordance with previous reports^22^,^23^,^29^,^31^, we observed preferential usage of *TRBV19* (72.2%), *TRBJ2-7* (31.1%) and *TRAJ42* (24.4%) genes in the analysed Flu MP_58-66_-specific CD8^+^ T clonotypes (Supplementary Fig. S1c). These findings endorse the approach taken to obtain antigen-specific TCR information.

**Figure 1:**
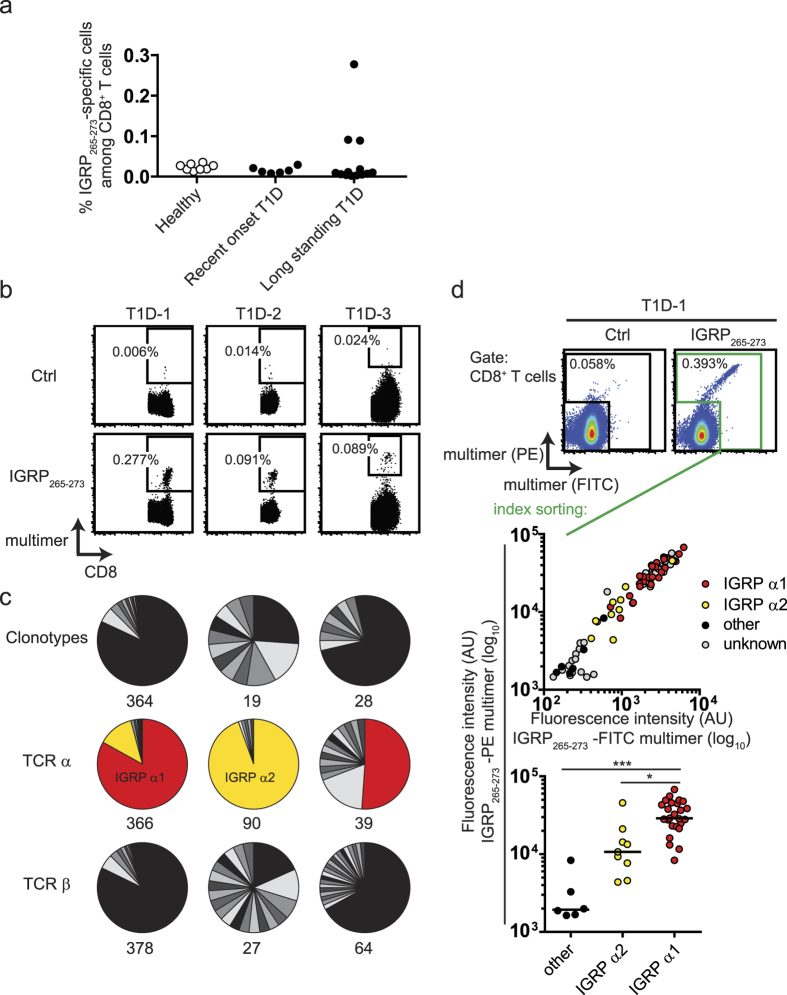
Identification and TCR repertoire analysis of IGRP_265-273_-specific CD8^+^ T cells. (**a**) Frequencies of multimer positive IGRP_265-273_-specific cells (y axis) among CD8^+^ T cells in HLA A*0201 healthy children (n = 8) age matched to children with recent onset of type 1 diabetes (n = 6), and adult patients with long standing type 1 diabetes (n = 13) were determined via flow cytometry. (**b**) Representative multimer staining FACS plots of PBMC samples of donors with type 1 diabetes and highest frequencies of IGRP_265-273_-specific CD8^+^ T cells in (**a**). PBMC samples were stained with HLA-A2 multimers loaded with control peptide (HLA-A2_140-149_; top row) or IGRP_265-273_ (bottom row). Plots show cells in the CD8 gate; 10^5^ (T1D-1, T1D-2) or 5 × 10^4^ (T1D-3) CD8^+^ T cell events are shown. (**c**) TCR repertoire analysis upon TCR α- and β-chain sequencing of IGRP_265-273_-specific CD8^+^ T cells isolated as single cells from T1D-1 (left), T1D-2 (middle) and T1D-3 (right). Each piece of pie charts represents cells with the same TCR α-chain and TCR β-chain combination (clonotypes; upper row), TCR α-chain (middle row) or TCR β-chain (bottom row). Numbers of analyzed cells are indicated. Shades of gray represent private clonotypes or TCR chains, colors indicate sharing among individuals. Note: numbers and pie charts of donor 1 comprise information of all three analyzed time points shown in Supplementary Fig. S3. (**d**) Combined multimer analysis and TCR sequencing via single cell index sorting. PBMC from T1D-1 were stained with PE and FITC HLA-A2 multimers loaded with either control peptide (HLA-A2_140-149_; top left) or IGRP_265-273_ (top right). Using index sorting, multimer binding cells were isolated as single cells for TCR sequencing. TCR α-chain sequencing information was subsequently attributed to the individual sorted cells and visualized using index sorting fluorescence intensity information (middle). Cells for which no TCR α-chain information was obtained are depicted in gray. The bottom plot compares PE-multimer fluorescence intensities (y axis) of cells expressing the dominant TCR α-chains IGRP α1 or IGRP α2, and other TCR α-chains. Lines indicate median values and significant differences between groups according to one-way ANOVA using Dunn’s multiple comparison test are marked (*P = 0.01–0.05; ***P < 0.001).

We proceeded to analyse CD8^+^ T cells with specificity against the type 1 diabetes autoantigen epitope, IGRP_265-273_. Consistent with a previous report^19^, we observed no difference in the frequency of CD8^+^ T cells that stained positive with multimers loaded with the HLA-A*0201 restricted peptide epitope IGRP_265-273_ between healthy controls (median frequency, 0.02% of CD8^+^ T cells) and children with recent onset of type 1 diabetes (0.01%; *P* = 0.11) or long-standing type 1 diabetes (0.01%; *P* = 0.06; Fig. 1a; Supplementary Table S2). However, three patients showed a prominent and distinct population of IGRP_265-273_-specific CD8^+^ T cells (T1D-1, T1D-2, T1D-3; Fig. 1b). The IGRP_265-273_-specific CD8^+^ T cells in at least two of these patients had an antigen-experienced memory phenotype, which was in contrast to a predominantly naive phenotype of IGRP_265-273_-specific CD8^+^ T cells in other patients tested and in healthy controls (Supplementary Fig. S2).

Paired TCR α- and β-chain sequences of 411 IGRP_265-273_-specific CD8^+^ T cells were obtained from patients T1D-1, T1D-2, and T1D-3. Dominant TCR α- and β-chain combinations or clonotypes, defined as a unique combination of TCR α- and β-chains, were observed in each of the three patients (Fig. 1c and Supplementary Table S1). Moreover, the dominant IGRP_265-273_-specific CD8^+^ T cell clonotypes persisted in samples taken more than 10 months apart from patient T1D-1 (Supplementary Fig. S3). Of note, the major TCR α-chains were shared among the three patients, paired with a number of different TCR β-chains (Fig. 1c and Supplementary Table S1). Interestingly, as revealed by combined index sorting of IGRP_265-273_ multimer-positive CD8^+^ T cells and TCR sequencing for T1D-1, those cells that expressed the dominant TCR α-chain detected in T1D-1 and T1D-3 (IGRP α1; depicted as red piece of pie charts in Fig. 1c) had the highest median fluorescence intensity in the IGRP_265-273_-specific multimer staining (Fig. 1d) as compared to the IGRP α2-containing cells (depicted as yellow piece of pie charts in Fig. 1c; *P* < 0.05) and cells with other TCR α-chains (*P* < 0.001).

Taken together, by analysing the TCR repertoires of IGRP_265-273_ directed CD8^+^ T cells from patients with type 1 diabetes, we identified dominant IGRP_265-273_–specific clonotypes that were persistent in sequential samples and common usage of IGRP_265-273_–specific CD8^+^ T cell-derived TCR α-chains between individuals.

### The TRAJ53 SGGSNYKLTF motif is frequent in HLA-A*0201 IGRP-specific clonotypes

**Figure 2:**
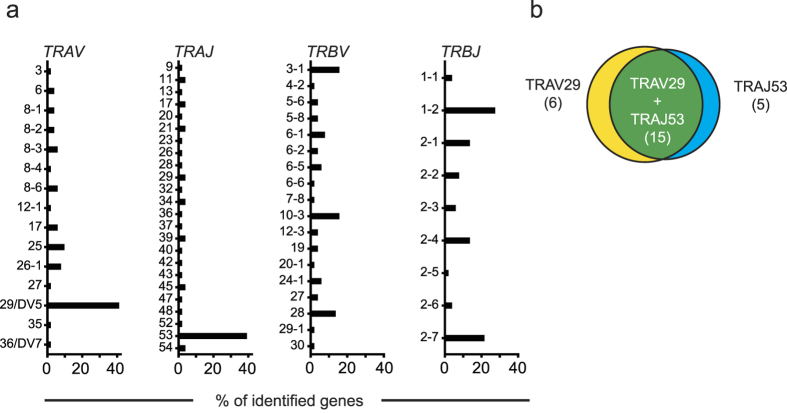
*TRAV29/DV5* and *TRAJ53* are frequent in IGRP_265-273_-specific CD8^+^ T cells. (**a**) TCR gene usage identified in IGRP_265-273_-specific CD8^+^ T cells. Frequencies (x-axis) of TRAV, TRAJ, TRBV and TRBJ genes used in unique clonotypes (n = 51, of which 48 are from patient-derived cells) are shown. (**b**) *TRAV29/DV5* and *TRAJ53* usage coincide. Venn-diagram of IGRP-specific clonotypes expressing *TRAV29/DV5* only (yellow), *TRAJ53* only (blue) or both (green).

Examining the paired TCR α- and β-chains of single cell-sorted IGRP_265-273_-specific CD8^+^ T cells, we identified the CDR3 motif SGGSNYKLTF in the TCR α-chain of the dominant clonotypes of patients T1D-1 (299 cells), T1D-2 (5 cells), and T1D-3 (20 cells), and in a further 60 of 94 patient derived IGRP_265-273_-specific CD8^+^ T cells for which we obtained TCR α- and β-chain information. In total, 384 (91.9%) of 418 analysed patient-derived IGRP-specific CD8^+^ T cells for which we obtained TCR α- and β-chain information contained this CDR3 motif (6 patients had cells with full clonotype information; see Supplementary Table S1). The motif corresponds to the *TRAJ53* gene, which together with *TRAV29/DV5*, are the predominantly used TRAJ and TRAV genes within all of the 48 identified patient-derived IGRP_265-273_-specific clonotypes (41.7% and 43.8%, respectively; Fig. 2a). Both *TRAJ53* and *TRAV29/DV5* genes were seen in 15 (31.3%) clonotypes (Fig. 2b), represented in 61 (14.6%) IGRP_265-273_-specific CD8^+^ T cells. Three clonotypes were identified in IGRP_265-273_-specific CD8^+^ T cells from healthy individuals. None of these had the SGGSNYKLTF motif in their TCR α-chain CDR3.

A part of the SGGSNYKLTF motif (GSNA/YKLT) was previously found in T lymphocytes isolated from pancreatic islets of NOD mice^32^,^33^,^34^, as well as in 2 of 53 CD4^+^ T cell clones isolated from islets of a HLA-A*0201 positive human patient with type 1 diabetes^35^. We, therefore, searched for the SGGSNYKLTF motif in our antigen-specific T cell database. Of the 51 IGRP_265-273_-specific CD8^+^ T cell clonotypes identified in both patients and controls, 19 (37.3%) had the SGGSNYKLTF motif. In comparison, 2 out of 90 (2.2%; *P* < 0.0001) of the TCR α-chains from Flu MP58-66-specific CD8^+^ T cell clonotypes, 0 out of 18 (0%; *P* = 0.0015) of the TCR α-chains from CMVpp65-specific CD8^+^ T cell clonotypes^24^, and 29 out of 1492 (1.9%, P < 0.0001) TCR α-chains from GAD65- or Tetanus toxoid-specific CD4^+^ T cell clonotypes analysed in previous studies^36^ had the SGGSNYKLTF motif arguing for an enrichment of this motif in IGRP-specific cells and against a general bias for this motif in antigen-specific T cells.

### Dominant IGRP_265-273_-specific CD8^+^ T cell TCR α-chain and SGGSNYKLTF motif frequencies in CD8^+^ T cell repertoires

TCRs raised against specific antigens are proposed as potential disease biomarkers^37^. We were, therefore, interested in determining whether any of the TCR α-chains identified in IGRP_265-273_ directed cells were associated with type 1 diabetes. We first applied next generation sequencing of the TCR α-chain repertoire to determine whether the approach could detect the dominant IGRP_265-273_-specific chains IGRP α1 and IGRP α2 in bulk CD8^+^ T cells from the original patients T1D-1, T1D-2, and T1D-3 (Supplementary Fig. S4). Consistent with the single cell TCR sequencing results (Fig. 1), we retrieved message for IGRP α1 exclusively within the libraries of donors T1D-1 and T1D-3. Message for the dominant IGRP α2 was detected within both the libraries of T1D-1 and T1D-2.

**Figure 3:**
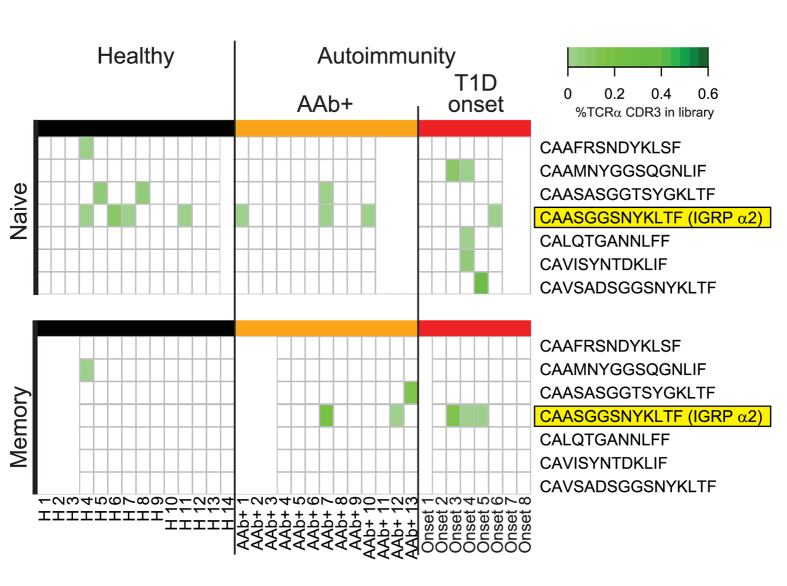
Screening for IGRP_265-273_-specific TCR sequences in bulk sorted naïve and memory CD8^+^ T cells using TCR α-chain NGS. Naïve (CCR7^+^CD45RA^+^) and memory (CCR7^+^CD45RA^−^, CCR7^−^CD45RA^+/−^) CD8^+^ T cells of healthy donors (n = 14) or donors with multiple islet autoantibodies (AAb+; n = 13) or recent onset type 1 diabetes (n = 8) were flow sorted from PBMC, their RNA extracted, individual cDNA libraries prepared and processed via next generation sequencing. TCR α-chain sequences were extracted and screened for those previously identified in IGRP_265-273_-specific single CD8^+^ T cells. TCR α-chain CDR3 region sequences retrieved from the donor libraries are listed on the right. Filled (green) boxes within the heatmaps indicate libraries in which the sequences were found, and shades of green visualize their abundance within the given libraries. White space holders in the heatmaps are shown if information was available only for the naïve or memory library.

We, therefore, performed TCR α-chain next generation sequencing on naïve and memory CD8^+^ T cells from a new set of HLA A*0201 positive donors comprising control children (n = 14), multiple islet autoantibody (AAb+; n = 13) positive children, and recent onset patients with type 1 diabetes (n = 8). Seven of the 45 TCR α-chains identified in IGRP_265-273_ multimer-sorted single cells were detected in either the naïve (7 TCR α-chains) or memory (3 TCR α-chains) CD8^+^ T cells from these individuals (Fig. 3). Within memory CD8^+^ T cells, IGRP α2, the dominant TCR α-chain shared by patients T1D-1 and T1D-2, was detected in none of 11 control children as compared to 2 (20%) of 10 autoantibody positive children and 3 (43%) of 7 recent onset type 1 diabetes patients (*P* = 0.042 controls vs patients). Moreover, IGRP α2 read frequencies were higher in the memory CD8^+^ T cell repertoires of patients as compared to controls (*P* = 0.026). No other differences were observed.

We next examined whether the SGGSNYKLTF motif is *per se* associated with type 1 diabetes (Supplementary Fig. S5). TCR α-chains that contained the SGGSNYKLTF motif were detected in all donors and their abundance in naïve or memory CD8^+^ T cells was not significantly increased in AAb+ (median, naive 0.77% and memory 0.89%) or recent onset type 1 diabetes patients (median, 1.06% and 0.63%) as compared to control children (median, 1.07% and 0.73%), arguing against a general association of TCRs containing this motif with disease.

### Dominant IGRP TCR α-chain expressing T cell clones specifically kill targets

**Figure 4:**
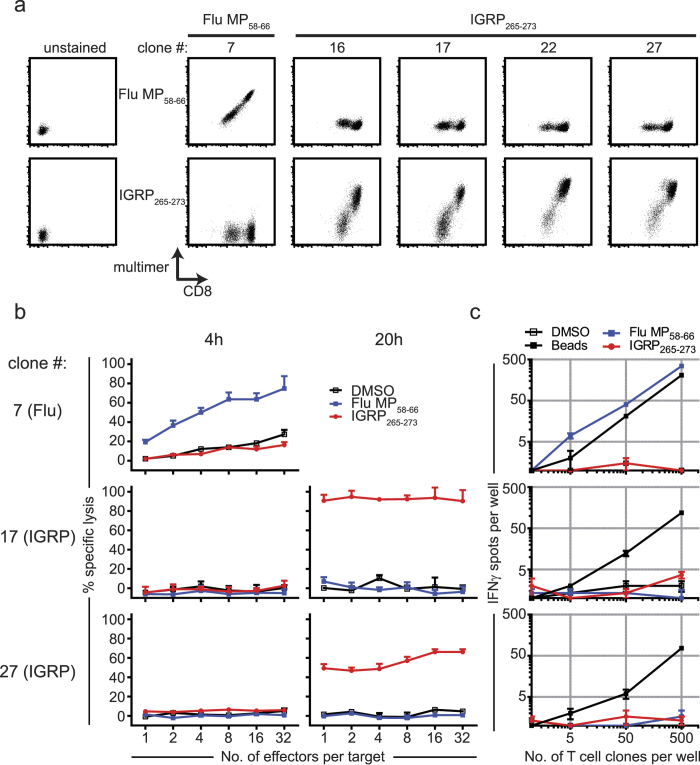
Functional characterization of IGRP_265-273_-specific CD8^+^ T cell clones. (**a**) IGRP_265-273_ (clones 16, 17, 22, 27) and Flu MP_58-66_ (clone 7) directed clones isolated from T1D-1 stained with anti-CD8 and HLA-A2 multimers loaded with Flu MP_58-66_ or IGRP_265-273_. Unstained samples served as controls (left column). Representative FACS plots of at least 3 independent experiments are shown. (**b**) IGRP-specific CD8^+^ T cell clones kill peptide loaded target cells. Flu MP_58-66_-specific clone 7 (top) and IGRP_265-273_-specific clones 17 (middle) and 27 (bottom) were titrated in a ^51^Cr release assay using K562/A*0201 cells as targets. Clones at increasing effector to target cell ratios (x axis) were stimulated with Flu MP_58-66_ peptide (blue), IGRP_265-273_ peptide (red) or solvent DMSO (black), and the percentages of specific lysis of target cells (y axis) were analysed after assay incubation for 4 h and 20 h. Representative data of three independent assays are shown. Data are presented as mean + SEM of triplicate wells. (**c**) IFNγ secretion by MP_58-66_ or IGRP_265-273_-specific CD8^+^ T cell clones. Titrated numbers of Flu MP_58-66_-specific (clone 7, top) and IGRP_265-273_-specific (clones 17 and 27, middle and bottom, respectively) clonal CD8^+^ T cells (x axis) were incubated in IFNγ ELISpot assays using K562/A*0201 cells for antigen presentation and stimulated with Flu MP_58-66_ peptide, IGRP_265-273_ peptide or control stimuli (solvent DMSO, anti-CD3/anti-CD28 coated beads). Data points in the analysis graphs show mean number of spots ± SEM of triplicate wells (y axis). Representative assays of three independent experiments are shown.

To further assess the peptide specificity of IGRP directed clonotypes containing the *TRAJ53* SGGSNYKLTF motif, we expanded IGRP_265-273_ directed single cells and Flu MP_58-66_ directed cells from patient T1D-1 to obtain CD8^+^ T cell clones. Clones 16 and 17 expressed the dominant IGRP directed TCR of T1D-1 (comprising the *TRAJ53*-encoded IGRP α1) and clones 22 and 27 had identical TCRs comprising the *TRAV29/DV5-TRAJ53*-encoded IGRP α2 (see Supplementary Table S3 for TCR sequence information for CD8^+^ T cell clones). Binding to peptide loaded HLA-A*0201 multimers was confirmed for each of these IGRP_265-273_ directed clones and a Flu MP_58-66_ directed clone (clone 7; Fig. 4a). IGRP_265-273_ directed clones 17 and 27 were able to mediate peptide-specific target cell lysis, albeit at a later time point than the Flu MP_58-66_ directed clone 7 (Fig. 4b). Unlike the Flu MP_58-66_-directed clone 7, the IGRP_265-273_-specific clones did not yield IFNγ spot formation when stimulated with IGRP_265-273_ peptide, despite strong responses to a polyclonal stimulus (Fig. 4c). The single cell gene expression profiles of expanded Flu MP_58-66_- and IGRP_265-273_ directed clones also differed. While cells from all clones expressed many genes typical of activated CD8^+^ T cells such as *GZMA* and *GZMB*, we noted higher expression of *GZMH (P* = 7 × 10^−8^), and less *TBX21 (P* = 2.9 × 10^−4^), *CD52 (P* = 0.0012) and *TNF (P* = 0.0073) in the IGRP_265-273_ directed clones as compared to the Flu-specific clone (Supplementary Fig. S6).

## Discussion

We applied single cell TCR α- and β-chain sequencing to assess the TCR repertoire of CD8^+^ T cells directed against IGRP_265-273_ as a model target epitope of type 1 diabetes. We identified dominant IGRP-specific clonotypes in patients that were persistent in sequential samples, detected sharing of dominant TCR α-chains among patients, and identified a motif that is overrepresented in IGRP_265-273_-specific CD8^+^ T cells as compared to T cells with other antigen specificities.

The low frequency of islet-antigen-specific CD8^+^ T cells in the peripheral blood of type 1 diabetes patients has thus far hampered a detailed analysis of their TCR repertoire. The overall low frequency of IGRP_265-273_-specific cells in samples from both type 1 diabetes patients and controls observed in this study is in agreement with previous reports^17^,^19^ that looked at frequencies and phenotypes of CD8^+^ T cells specific for IGRP and additional autoantigen-specific epitopes, although one of these reports suggested increased frequencies of IGRP-specific CD8^+^ T cells in patients as compared to controls^17^. The identification of three patients with expanded populations of memory CD8^+^ T cells specific to IGRP_265-273_ allowed us to isolate and analyse a large number of IGRP_265-273_-specific cells. Although it is possible that our findings on TCR from these patients are not representative of all subjects, it is remarkable that we found sharing of TCR α-chains between the patients and that sharing was marked by a *TRAJ53* encoded motif. These findings, as well as the impressive correlation between TCR α-chain and multimer staining intensity, the persistency of expanded clonotypes over time, and the ability of clones derived from multimer-positive CD8^+^ T cells to antigen-specifically lyse target cells speak for *bona fide* antigen-specific cells. Further supporting the methodological approach, we find similar TCR gene usage for Flu MP_58-66_-specific CD8^+^ T cells to that reported in previous reports^22^,^23^,^29^,^30^ and identical TCR chain CDR3 sequences and public motifs contained in them^38^.

Selection of antigen-specific T cells in our study was based upon multimer staining. As we demonstrated, different TCRs varied in their multimer binding intensities, suggesting high and low affinity TCRs. Studies in the NOD mouse have demonstrated that TCR avidity against IGRP is relevant to disease stage, with higher avidity clones marking a feature of disease progression^39^. Studies in the NOD mouse argue against IGRP as a primary target in the autoimmune disease process^40^ and describe a predominant presence of IGRP directed CD8^+^ T cells in later stages of disease^28^. It is, therefore, potentially interesting to find expansion of high affinity IGRP_265-273_-specific TCRs in the patient with long-standing type 1 diabetes. We did not, however, study sufficient numbers of patients to conclude that the expanded population with a memory phenotype is related to disease duration or accumulation over time.

The repertoire of IGRP-specific cells was highly enriched in TCR α-chains comprising the motif SGGSNYKLTF that is encoded by the *TRAJ53* gene, especially when combined with the *TRAV29/DV5* gene. Interestingly, this motif, and a highly similar motif encoded by *Traj42* (SGGSNAKLTF), has been identified previously in autoreactive and pancreatic islet derived T cells in NOD mice^32^,^34^, including CD8^+^ T cells reactive to Igrp peptide^33^ and CD4^+^ T cells reactive to the Insulin peptide B:9-23^41^. Moreover, TCRs targeting the insulin B:9–23 peptide presented by the I-A^g7^ MHC class II molecule frequently use the Vα gene segment *Trav5D-4* rearranged to the Jα gene segments *Traj53* and *Traj42*^42^,^43^. Thus, islet antigen reactive T cells in NOD mice and man appear to have a recurring preference for certain TCR α-chain genes. Such selection, if true, could be explained by structural similarities across multiple epitope/MHC targets.

Finally, in an attempt to identify molecular markers of type 1 diabetes, we identified one TCR α-chain that, in memory CD8^+^ T cells, was restricted to patients with type 1 diabetes or pre-type 1 diabetes. The association was not striking, but suggests that it may be possible to identify panels of clonotypes that mark type 1 diabetes-relevant CD8^+^ T cell activity. A limitation is that we did not perform next generation sequencing for the TCR β-chain. Nevertheless, our findings support the use of approaches similar to those used in this study to extend the knowledge of beta cell antigen-specific TCR repertoires. Moreover, it is possible that both the expansion of autoreactive T cells we observed for IGRP and an apparent preference for certain TCR α-chain genes in responses to different autoantigens reflects cross-reactivity to unknown non-self antigens^44^, which may be favoured by selection of T cells that bear TCRs with a moderate ability to cross-react with multiple peptides^45^.

## Methods

### Subjects

Samples were obtained from 75 individuals who had the HLA-A*0201 allele (Supplementary Table S2). These included 27 patients with type 1 diabetes (16 female, 11 male; median age at blood sampling: 22.9 years; range, 5.3–45.9 years), 14 of whom had a diabetes duration of less than one year (median disease duration: 0.04 years; range, 0.01–0.94 years) and 13 patients with a type 1 diabetes duration of more than one year (median disease duration: 14.33 years; range, 1.1–41.5 years); 13 non-diabetic autoantibody positive relatives of patients with type 1 diabetes (3 female, 10 male; median age: 11.6 years; range, 3.8–18.7 years) who had autoantibodies against at least two of four islet antigens (Insulin, GAD65, ZnT8, IA-2), and 35 healthy controls (20 female, 15 male; median age 14.3 years, range, 8.3–63.0 years). All methods were performed in accordance with relevant guidelines and regulations. Samples were collected with informed consent as part of studies that were approved by the ethical committees of the Technische Universität München (No. 2149/08) or Bavaria, Germany (Bayerische Landesaerztekammer, Nr.08043).

### Peptides

Peptides used in this study were purchased from Mimotopes, Australia at >95% purity, as confirmed using HPLC and mass spectrometry.

### Cell staining and flow cytometry

Cells were stained using the following monoclonal antibody-fluorochrome combinations: CD4-APC (clone SK3), CD8-APC-Cy7 (clone SK1), CD14-APC (clone M5E2), CD19-APC (clone HIB19), CD56-APC (clone B159) from BD Pharmingen; CD8a-eFluor450 (clone SK1), CD8a-PE (clone SK1), CD45RA-eFluor605NC (clone HI100) from eBioscience; CD16-APC (clone 3G8), CD197(CCR7)-Brilliant Violet 710 from BioLegend; CD335-APC (clone 9E2) from Miltenyi Biotech. 7-Aminoactinomycin D (7AAD, BD Pharmingen) or Sytox Blue (Invitrogen) were used to exclude dead cells. FITC or PE labeled HLA-A*0201 multimers loaded with either Flu MP_58-66_ (GILGFVFTL), IGRP_265-273_ (VLFGLGFAI) or a control peptide of the HLA-A2 protein (HLA-A2_140-149_; YAYDGKDYIA) were purchased from Immudex (Copenhagen, Denmark). Cells were acquired on a Becton Dickinson ARIA II or ARIA III flow sorter with FACS Diva software (for index sorting experiments, FACS Diva 8 software was used) and analysed using FlowJo software (FlowJo LLC Ashland, OR, USA). Unless stated otherwise, cells were stained in PBS supplemented with 1% pooled human AB serum for 30 min on ice followed by at least two washing steps using the same buffer and staining with dead cell marker before cell analysis. Multimer staining was carried out on thawed or fresh PBMC samples after overnight resting at 37 °C/5% CO_2_ in X-VIVO 15 supplemented with 5% pooled human AB serum. Cells were resuspended in PBS supplemented with 5% FBS and 50 nM dasatinib and incubated for 30 min at 37 °C. 2 × 10^6^ cells were stained with multimers for 10 min at room temperature in the same buffer followed by an additional 20 min incubation at 4 °C in the presence of the respective antibodies. Cells were washed twice with PBS/5% FBS and incubated with 7AAD or Sytox Blue for 10 min immediately before flow cytometry. Gating strategies for the analysis and cell sorting of multimer stained samples are exemplarily shown in Supplementary Fig. S7. Gates for antigen-specific CD8^+^ T cell frequency analysis were based on staining using the control multimers or, when available, according to staining data of PBMC spiked with the respective antigen-specific CD8^+^ T cell clone.

### TCR sequencing

RT-PCR amplification and sequencing of expressed TCR α- and β-chain genes from single CD8^+^ T cells, as well as subsequent cloning of PCR fragments, were performed as previously described^46^. Analysis of TCR α- and TCR β-chain sequences and junction peptide amino acid sequence extraction was conducted with reference to the International ImMunoGeneTics Information System database^47^. Obtained junction peptides were then analyzed using KNIME 2.11.2^48^.

The TCR α-chain repertoire of 2 × 10^5^ flow-sorted total, naïve (CD45RA^+^CCR7^+^) or memory (CD45RA^+/−^CCR7^−^ and CD45RA^−^CCR7^+^) peripheral blood derived CD8^+^ T cells from patients and controls was analysed using next generation sequencing as previously described^36^. Libraries containing < 0.5 Mio reads were excluded. TCR CDR3 region sequence extraction and PCR error correction was performed with MiTCR^49^. All additional sequence analysis was conducted in KNIME 2.11.2.

### Cloning of antigen specific CD8^+^ T cells

Flu MP_58-66_- and IGRP_265-273_-specific CD8^+^ T cell clones were propagated from multimer positive single cells according to a previously described protocol^5^. In brief, purified CD8^+^ T cells were stained with the respective multimers and viable multimer positive CD8^+^ T cells were flow-sorted and seeded at 1 cell/well in 96-well plates containing 10^5^ irradiated allogeneic PBMCs per well in X-VIVO15 supplemented with 5% Cellkine (Zeptometrix Corp., NY, USA), IL-7 (10 ng/ml), IL-15 (0.1 ng/ml) and PHA-M (5 μg/ml). CD8^+^ T cell clones were then stimulated every 14 days at a 1:5 clone to feeder ratio (or smaller in the initial expansion phase, i.e. until a 1:5 ratio per well of a 96 well plate was achieved) with irradiated allogeneic PBMCs that alternately were used either in combination with PHA-M as described above or were preloaded with peptide (FluMP_58-66_ or IGRP_265-273_; 10 μg/ml).

### Cytotoxicity assay

Cytotoxicity of propagated clones was analysed by radioactive ^51^Cr release assays. HLA-A*0201 expressing K562 cells (K562/A*0201^50^; kindly provided by Prof. Thomas Wölfel; Johannes Gutenberg Universität, Mainz, Germany) were used as target cells. Briefly, 5 × 10^3 51^Cr labelled target cells were seeded into V-shaped 96 well plates followed by effector cells (CD8^+^ T cell clones) in varying ratios. Assays were conducted in a final volume of 200 μl /well in X-VIVO15 medium supplemented with 5% AB serum, IL-7 (10 ng/ml) and IL-15 (0.1 ng/ml). Assays were conducted in triplicate and cultures were incubated for 20 h at 37 °C/5%CO_2_ in the presence of FluMP_58-66_ or IGRP_265-273_ peptides (10 μg/ml) or solvent (DMSO) control. After 4 or 20 hours incubation, 25 μl of supernatant from each well was transferred into 96 well sample plates containing 150 μl scintillation liquid per well. After 5 minutes shaking at room temperature ^51^Cr release was measured with a scintillation counter (1450 MicroBeta TriLux, Perkin Elmer). Maximum and spontaneous ^51^Cr release was determined from cells treated with lysis buffer or assay medium, respectively. Specific cytotoxicity was calculated using the formula: % specific release = (experimental–spontaneous release) × 100/(maximum–spontaneous release).

### ELISpot assay

ELISpot assays on CD8^+^ T cell clones were performed as previously described^16^ using K562/A*0201 cells for antigen presentation. In brief, 96-well polyvinylidine fluoride plates (Merck Millipore, Darmstadt, Germany) were coated overnight with an IFNγ directed antibody (U-CyTech biosciences, Utrecht, The Netherlands). Plates were subsequently washed and blocked with AIM-V plus 10% AB serum. Assays were carried out with 5 × 10^4^ K562/A*0201 cells/well in a 200 μl/well format using AIM-V medium supplemented with 0.5 ng/ml IL-7. Titrated numbers of viable CD8^+^ T cell clones (5–500 cells) were flow-sorted directly into the wells and incubated for 20–24 h in the presence of peptide (IGRP_265-273_ or Flu MP_58-66_; 10 μg/ml) or solvent (DMSO). Following removal of cells, IFNγ secretion was visualized as described previously^51^,^52^ using biotin-conjugated anti-IFNγ detector antibody (U-CyTech biosciences, Utrecht, The Netherlands), alkaline phosphatase-conjugated ExtrAvidin, and Sigmafast 5-bromo-4-chloro-3-indolyl phosphate/nitro blue tetrazolium (BCIP/NBT) tablets (Sigma-Aldrich Chemie GmbH, Taufkirchen, Germany). Spots were counted using a Bioreader 5000 (BioSys, Karben, Germany) and mean ± SEM values of at least triplicate wells calculated.

### Statistical analysis

Frequencies of multimer directed cells between groups were compared using the Mann Whitney U test. Fluorescence intensity values of index-sorted cells with different TCR α-chains were compared using one-way ANOVA with Dunn’s multiple comparison test. TCR α CDR3 motif frequencies between antigen-specific T cell clonotypes were compared using Fisher’s exact test. The presence or absence of TCR α-chain sequences in next generation sequencing information was compared between groups using Fisher’s exact test. Read frequencies were compared using Mann-Whitney U test. Statistical analysis was performed using GraphPad prism (version 5.04) or R (version 3.2.5).

## Additional Information

**How to cite this article:** Fuchs, Y. F. *et al*. CD8^+^ T cells specific for the islet autoantigen IGRP are restricted in their T cell receptor chain usage. *Sci. Rep.* **7**, 44661; doi: 10.1038/srep44661 (2017).

**Publisher's note:** Springer Nature remains neutral with regard to jurisdictional claims in published maps and institutional affiliations.

## Supplementary Material

Supplementary Figures and Tables
