# Supplementary material for: CD8+ T cells specific for the islet autoantigen IGRP are restricted in their T cell receptor chain usage
Source: Sci Rep. 2017 Mar 16;7:44661. doi: 10.1038/srep44661 (PMC5353542; doi:10.1038/srep44661)
Supplement: Supplementary Figures and Tables [file srep44661-s1.pdf]

Online Supplementary Information

CD8$^+$ T cells specific for the islet autoantigen IGRP are restricted in their T cell receptor chain usage

Yannick F. Fuchs, Anne Eugster, Sevina Dietz, Christian Sebelefsky, Denise Kühn, Carmen Wilhelm, Annett Lindner, Anita Gavrisan, Jan Knoop, Andreas Dahl, Anette-G. Ziegler and Ezio Bonifacio

Supplementary Figures:

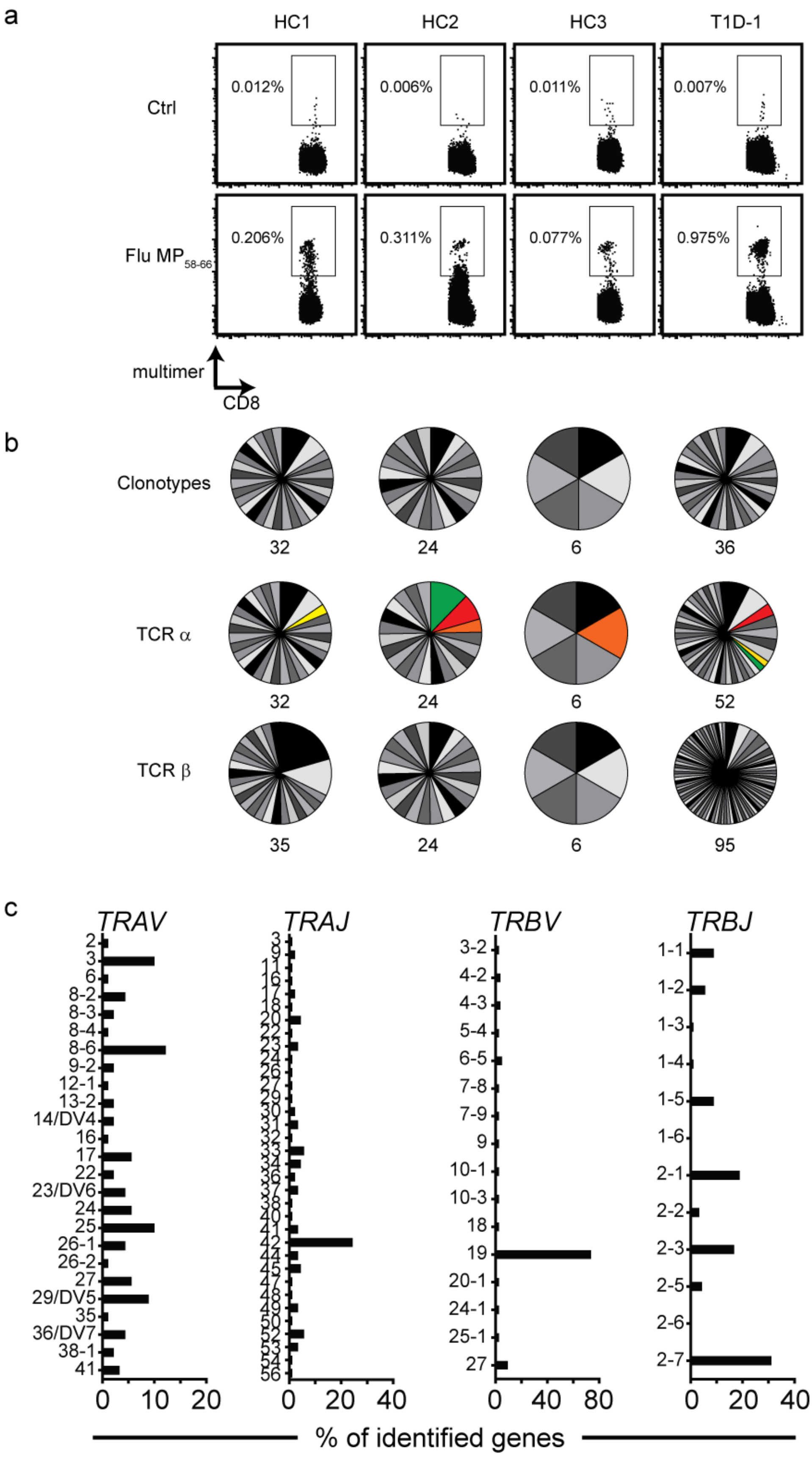

Supplementary Fig. S1: Identification and TCR repertoire analysis of Flu MP_{58-66}-specific $CD8^+$ T cells. (**a**) Representative FACS plots of PBMC samples of 3 healthy and one donor with long standing type 1 diabetes (T1D-1) stained with HLA-A2 multimers loaded with control peptide ($HLA\text{-}A2_{140-149}$; top row) or Flu MP_{58-66} (bottom row). Cells are gated on CD8 and 10^5 $CD8^+$ T cells are depicted in each plot. (**b**) TCR repertoire analysis upon TCR α- and TCR β-chain sequencing of Flu MP_{58-66} specific $CD8^+$ T cells isolated as single cells from samples shown in A. Pieces of pie charts represent cells with the same TCR α-/TCR β-chain combination (clonotypes; upper row), TCR α-chain (middle row) or TCR β-chain (middle row). Numbers of analyzed cells are indicated. Shades of gray represent private clonotypes or TCR chains, colors indicate sharing among individuals. (**c**) Frequencies of TRAV, TRAJ, TRBV and TRBJ genes detected in unique Flu MP_{58-66} specific clonotypes (n=90) identified in donors shown in A. TRAV, TRAJ, TRBV and TRBJ gene analysis reveals preferential usage of *TRBV19* and *TRBJ2-7* in TCR β-chains and *TRAJ42* in TCR α-chains of Flu MP_{58-66} specific $CD8^+$ T cells.

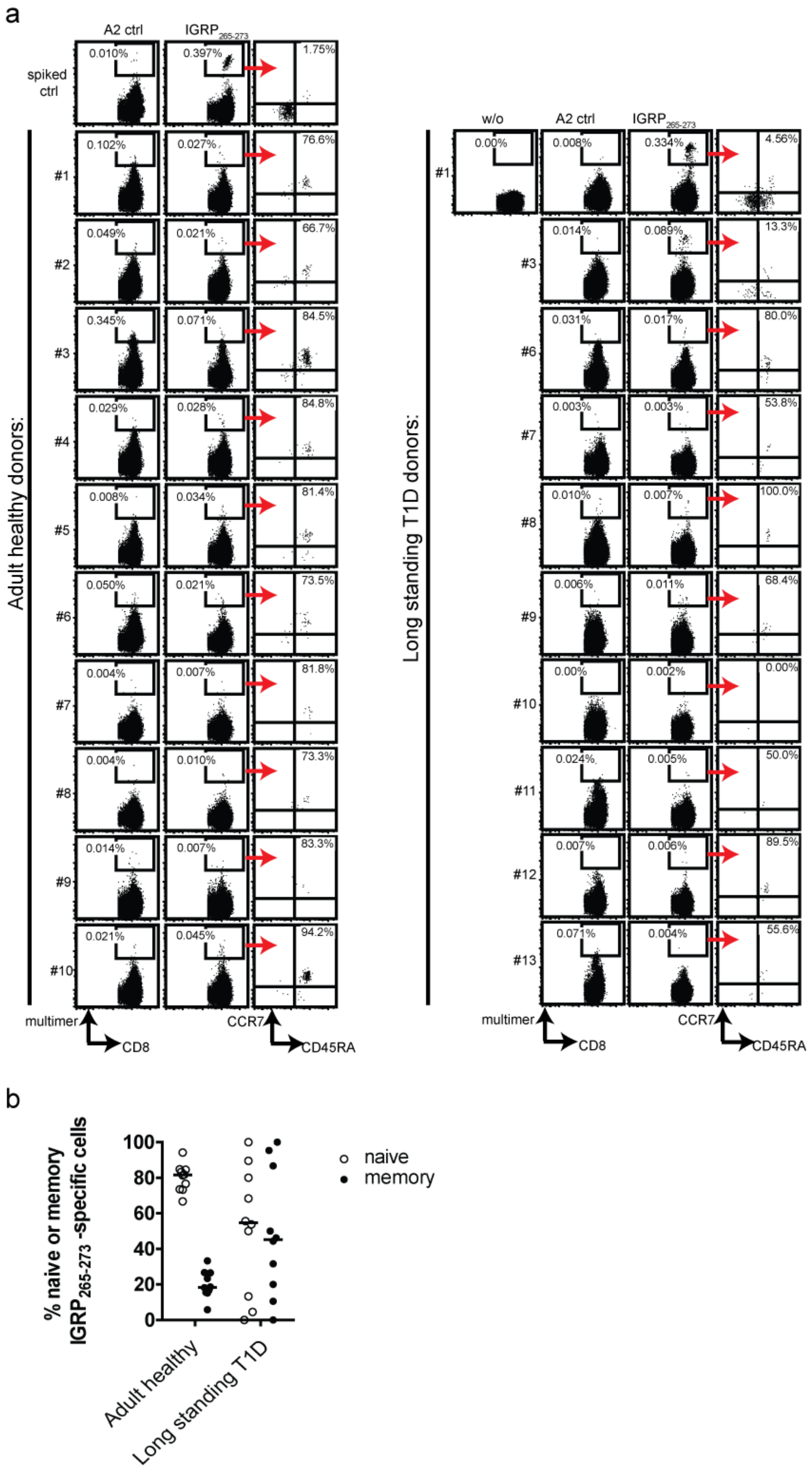
a
A2 ctrl
IGRP265-273
spiked ctrl
Adult healthy donors:
#1
#2
#3
#4
#5
#6
#7
#8
#9
#10
multimer
CD8
CCR7
CD45RA
w/o
Long standing T1D donors:
b
% naive or memory IGRP265-273 -specific cells
Adult healthy
Long standing T1D
naive
memory

Supplementary Fig. S2: Phenotypic characterization of IGRP$_{265\text{-}273}$-specific CD8$^+$ T cells in healthy adults and patients with long standing type 1 diabetes. (**a**) Analysis of CCR7 and CD45RA expression on IGRP$_{265\text{-}273}$-specific CD8$^+$ T cells in healthy donors (n=10) and patients with long standing type 1 diabetes (n=10). PBMC were stained without (w/o) or with either control peptide (HLA-A2$_{140\text{-}149}$) or IGRP$_{265\text{-}273}$ loaded multimers. Staining of PBMC samples spiked with IGRP$_{265\text{-}273}$ specific CD8$^+$ T cell clones served as controls (upper row). Plots of multimer stainings show cells in the CD8 gate and at least 5 x 10^4 events are shown. Plots of CCR7/CD45RA expression show all events of the ancestor gate and frequencies of naïve (CD45RA$^+$CCR7$^+$) cells are indicated. For patients with long standing type 1 diabetes representative FACS plots of donors with high (T1D-1 and T1D-3) and low frequencies (T1D-6-13) of IGRP$_{265\text{-}273}$ specific CD8$^+$ T cells are shown. (**b**) Data graph summarizing the frequencies of naïve (CCR7$^+$CD45RA$^+$; open symbols) and memory (CCR7$^+$CD45RA$^-$,CCR7$^-$CD45RA$^{+/-}$; filled symbols) IGRP$_{265\text{-}273}$-specifc CD8$^+$ T cells of adult healthy donors and patients with long standing type 1 diabetes shown in (a).

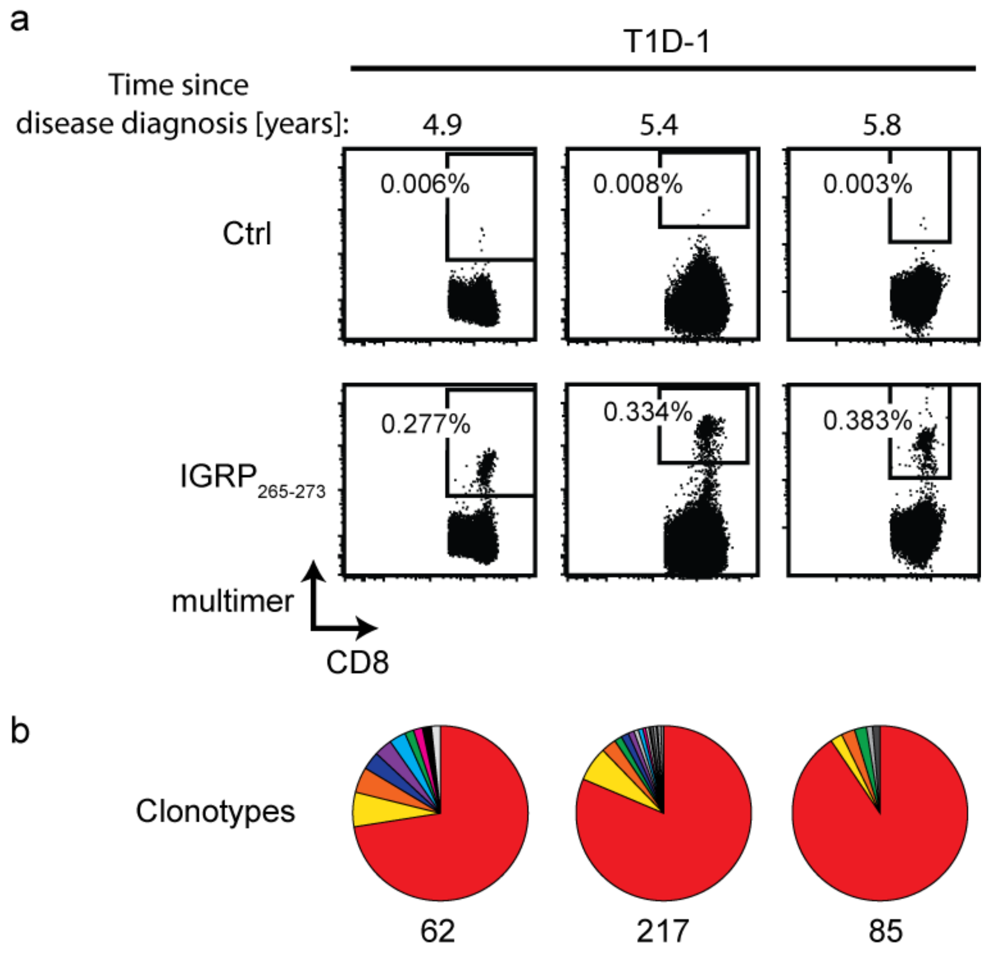

Supplementary Fig. S3: Persistence of IGRP$_{265\text{-}273}$ specific CD8$^+$ T cells and clonotypes in T1D-1. (**a**) Representative FACS plots of multimer stainings performed on PBMC samples taken at three independent timepoints. PBMC samples were stained with HLA-A2 multimers loaded with control peptide (HLA-A2$_{140\text{-}149}$; top row) or IGRP$_{265\text{-}273}$ (bottom row). Plots show 10^5 cells in the CD8 gate. (**b**) TCR repertoire analysis upon TCR α- and β-chain sequencing of IGRP$_{265\text{-}273}$ specific CD8$^+$ T cells isolated as single cells from samples of three individual timepoints. Pieces of pies represent distinct clonotypes and same colors represent same clonotypes. Numbers of cells for which parallel TCR α- and β-chain information was obtained for each individual timepoint are indicated.

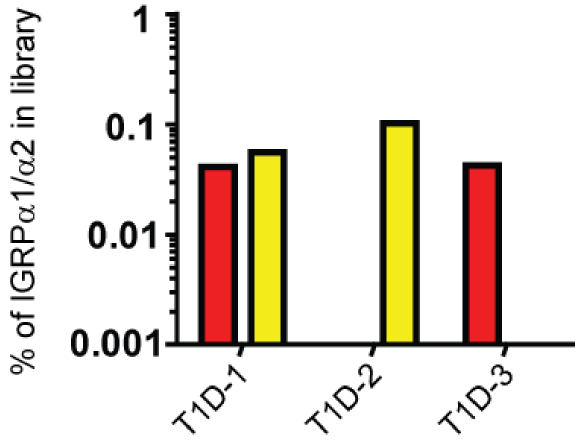

Supplementary Figure S4: Retrieval of IGRP α1 and IGRP α2 via TCR α-chain next generation sequencing. cDNA libraries of bulk sorted $CD3^{+}CD8^{+}$ T cells from donors T1D-1, T1D-2, T1D-3 were screened for message of the dominant $IGRP_{265\text{-}273}$-specific TCR α-chains IGRP α1 and IGRP α2 shown in Figure 1C (and depicted in red and yellow). Bars in the data graph represent frequencies (y axis) of the respective messages among all TCR α-chain messages in a given library.

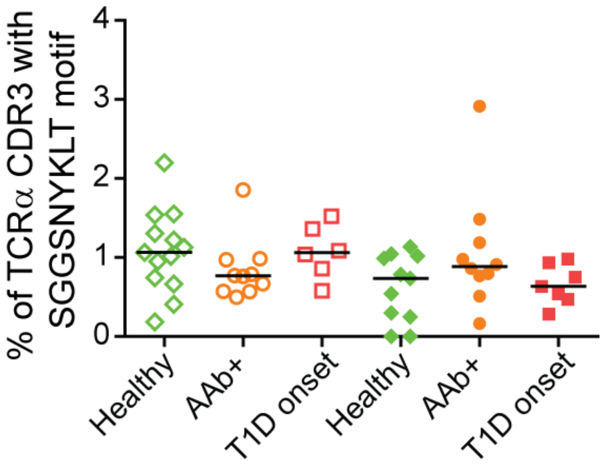

Supplementary Fig. S5: Next generation sequencing read frequencies of TCR α-chain CDR3 harboring the SGGSNYKLT motif in $CD8^{+}$ T cell libraries. Naïve ($CCR7^{+}CD45RA^{+}$; open symbols) and memory ($CCR7^{+}CD45RA^{-}$,$CCR7^{-}CD45RA^{+/-}$; filled symbols) $CD8^{+}$ T cells of healthy donors (n=14, green symbols) or donors with multiple islet autoantibodies (AAb+; n=13; orange symbols) or recent onset type 1 diabetes (n=8; red symbols) were flow sorted from PBMC, their RNA extracted, individual cDNA libraries prepared and processed via next generation sequencing. TCR α-chain sequences were extracted and screened for those harboring the SGGSNYKLT motif. Frequencies of motif containing sequences in individual libraries are shown. Lines indicate median values.

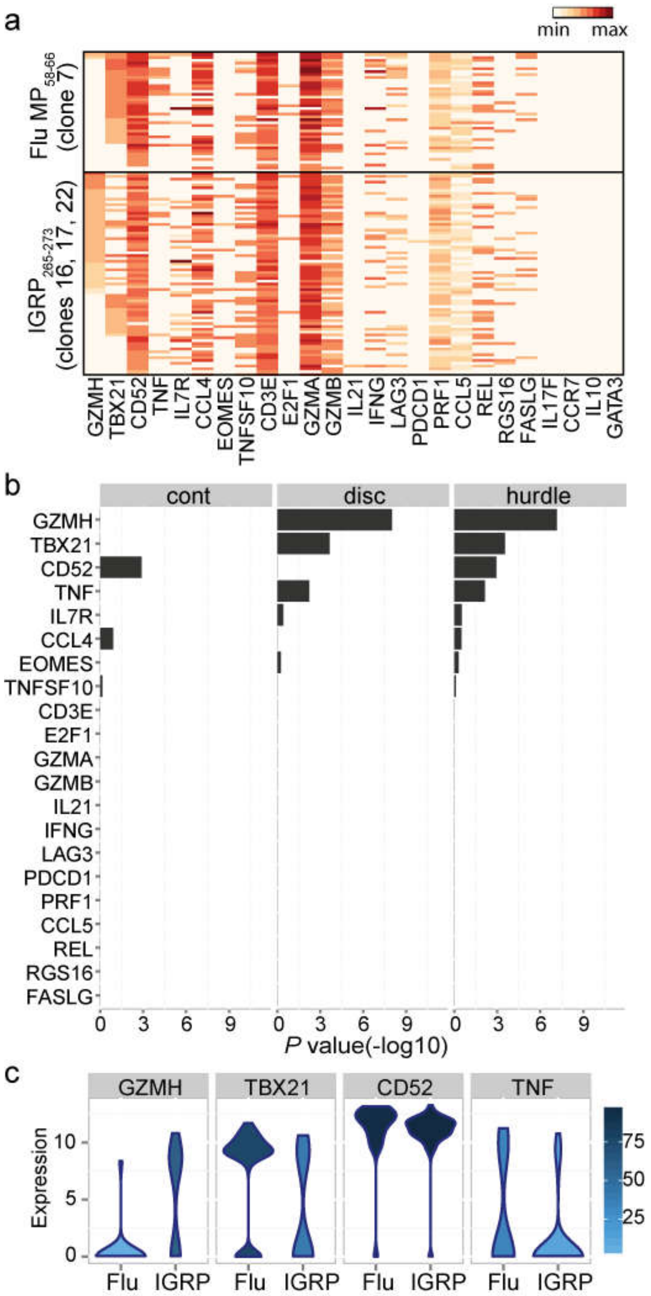

Supplementary Fig. S6: Gene transcription profiles of Flu MP$_{58-66}$ and IGRP$_{265-273}$ specific CD8^{+} T cell clones. Clones were single cell-sorted and processed for multi-parameter gene expression analysis as described in supplementary methods below. The profiles for 42 Flu MP$_{58-66}$-specific cells of clone 7 and 72 IGRP$_{265-273}$-specific cells of clones 16, 17, and 22 are shown. (**a**) Heatmaps showing expression profiles. Genes are ordered left to right according to their overall significance for comparisons between cells from the two groups using the Hurdle model. Each row represents an individual cell. Cells are ordered according to their expression of the genes *GZMH*, *TBX21,CD52 and TNF* (**b**) Bar chart showing p-values for gene expression comparisons between FluMP$_{58-66}$- and IGRP$_{265-273}$-specific CD8^{+} T cell clones using a continuous (left panel), discontinuous (middle) and the Hurdle (right) model. (**c**) Violin plots showing the distribution of Ct expression values for genes differentially expressed (P< 0.01) in Flu MP$_{58-66}$- and IGRP$_{265-273}$-specific cells in (A). The proportion of cells expressing the gene ranges from light blue (0%) to dark blue (100%).

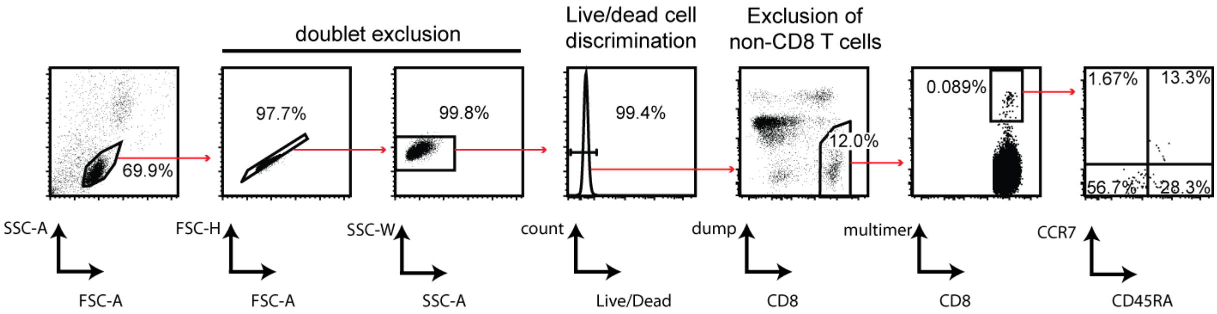

Supplementary Fig. S7: Gating strategy for the analysis and single cell sorting of antigen-specific CD8^{+} T cells. Representative FACS plots for the gating of multimer positive CD8^{+} T cells and the analysis of CCR7 and CD45RA expression are shown. Forward and side scatter channels were used to gate on lymphocytes and to exclude doublet cells. Live/Dead cell marker staining was used to gate on viable cells. Dump marker (CD4, CD14, CD16, CD19, CD56, CD335) were used to simplify gating on CD8^{high} T cells and subsequently multimer positive cells.

Supplementary Table S1: CD8$^+$ T cell clonotypes and TCR chains identified in multimer isolated antigen-specific single cells. TCR sequencing information of the CDR3 region retrieved from 775 antigen-specific CD8$^+$ T cells is listed. Rows are ordered according to the peptide antigen the cells were directed against, the individuals they were obtained from, the number of cells detected with a specific TCR information (enriched: information retrieved from more than one cell; single: information retrieved from one cell) and the completeness of TCR information, i.e paired TCRα/ TCRβ information followed by TCRα information only, TCRβ information only. TCR names are given to unique TCRα/ TCRβ pairs (clonotypes). TCRα and TCRβ CDR3 names are given to unique TCRα and TCRβ CDR3 sequences, respectively. Identical TCR CDR3 sequences present in several rows are marked (colored cells), TCR TRAV, TRAJ, TRBV and TRBJ genes used in more than 20 percent of clonotypes directed against the Flu MP_{58-66} or $IGRP_{265-273}$ antigen are marked with colored font. Inter-individual sharing of sequences or previous description of sequences by others[1-5] are remarked under comments. N/A=sequence information not available; T1D=type 1 diabetes patient; HC=healthy control donor; RO=recent onset

Please note: For facilitated extraction and further use of the sequence data an Excel-file of Supplementary Table S1 can be provided by the authors on request.

Antigen	Donor	TCR name (clonotype)	TRBV	TRBJ	TRBD	TCRβ CDR3 sequence	TCRβ CDR3 name	TRAV	TRAJ	TCRα CDR3 sequence	TCRα CDR3 name	Number cells	Comments
	HC1											Enriched	
FluMP58-66		FLUMP58-66_HC1_1	19*01	2-7*01	1*01	CASSIGDTSYEQYF	**FLU β1**	8-2*01	41*01	CVVSPAGYALNF	FLU α1	3	
		FLUMP58-66_HC1_2	19*01	2-7*01	1*01	CASSIGDTSYEQYF	**FLU β1**	8-2*01	41*01	VL*VLPG#ALNF	FLU α2	2	
			19*01	2-7*01	1*01	CASSIGDTSYEQYF	**FLU β1**	NA	NA	NA		2	
												Single	
		FLUMP58-66_HC1_3	19*01	2-7*01	1*01	CASSIGDTSYEQYF	**FLU β1**	16*01	30*01	CALRDDKIIF	FLU α3	1	
		FLUMP58-66_HC1_4	19*01	1-1*01	2*01	CASSIGGGQNTEAFF	FLU β2	26-1*01	16*01	CIVIHLSDGGQKLLF	FLU α4	1	
		FLUMP58-66_HC1_5	19*01	1-2*01	2*01	CASLGGGYPF	FLU β3	3*01	49*01	CAVRDMRVNGGNQFYF	FLU α5	1	
		FLUMP58-66_HC1_6	19*01	1-3*01	1*01	CASSTSSGNTIYF	FLU β4	24*01	20*01	CAFTNINDYKLSF	FLU α6	1	
		FLUMP58-66_HC1_7	19*01	1-5*01	1*01	CASSDGQPQHF	FLU β5	8-6*01	34*01	CAVND#NTDKLIF	FLU α7	1	
		FLUMP58-66_HC1_8	19*01	1-5*01	1*01	CASSTQGSQPQHF	FLU β6	8-6*02	31*01	CAVSDPAGNARLMF	FLU α8	1	
		FLUMP58-66_HC1_9	19*01	2-1*01	2*01	CASPPLGGGYNEQFF	FLU β7	24*01	44*01	CASPEIKTGPARKLTF	FLU α9	1	
		FLUMP58-66_HC1_10	19*01	2-1*01	1*01	CASSIVRGLYNEQFF	FLU β8	25*01	53*01	CAGLGGT#GGSNYKLTF	FLU α10	1	
		FLUMP58-66_HC1_11	19*01	2-1*01	NA	CASSIYGGNEQFF	FLU β9	24*01	31*01	CAQTRNNNARLMF	FLU α11	1	
		FLUMP58-66_HC1_12	19*01	2-1*01	2*01	CASGMIAGGLNERFF	FLU β10	29/DV5*01	20*01	VQQARNDYKLSF	FLU α12	1	
		FLUMP58-66_HC1_13	19*01	2-7*01	2*01	CASSFRSPNERNF	FLU β11	6*03	38*01	CAPQTRRLATTVS*#W	FLU α13	1	
		FLUMP58-66_HC1_14	19*01	2-7*01	1*01	CASSIRAAYEQYF	**FLU β12**	27*01	37*01	CAGESGNTGKLIF	FLU α14	1	TCRβ CDR3 previously described by: Valkenburg et al., 2016
		FLUMP58-66_HC1_15	19*01	2-7*01	1*01	CASSIRAAYEQYF	**FLU β12**	8-6*02	24*02	CAVSGPS#TDSWGKLQF	FLU α15	1	TCRβ CDR3 previously described by: Valkenburg et al., 2016
		FLUMP58-66_HC1_16	19*01	2-7*01	1*01	CASSIRAAYEQYF	**FLU β12**	3*01	42*01	CAVSYGGSQGNLIF	**FLU α16**	1	TCRα CDR3 shared with T1D-1; TCRβ CDR3 previously described by: Valkenburg et al., 2016
		FLUMP58-66_HC1_17	19*01	2-7*01	1*01	CASSTRSAYEQYF	FLU β13	8-6*02	17*01	CAVSAAX#AGNKLTF	FLU α17	1	
		FLUMP58-66_HC1_18	19*01	2-7*01	2*01	CASRTRPAYERDF	FLU β14	8-6*02	17*01	CAVSA#KAAGNKLTF	FLU α18	1	
		FLUMP58-66_HC1_19	19*01	2-7*01	2*01	CASSIVASGGRYF	FLU β15	25*01	54*01	CAGLGIIQGAQKLVF	FLU α19	1	
		FLUMP58-66_HC1_20	19*02	2-1*01	2*01	CASGRAGGPGNEQSF	FLU β16	8-6*02	48*01	GDEKLTF	FLU α20	1	
		FLUMP58-66_HC1_21	20-1*01	2-1*01	2*01	CSASLLASTSGGAGNEQFF	FLU β17	26-1*03	41*01	CIVRV#NSNSGYALNF	FLU α21	1	
		FLUMP58-66_HC1_22	27*01	1-1*01	1*01	CASTRGVGTEAFF	FLU β18	17*01	9*01	CATVPNTGGFKTIF	FLU α22	1	
		FLUMP58-66_HC1_23	27*01	2-7*01	1*01	CASGLRGPYERNF	FLU β19	3*01	18*01	CAVRDPDRGSTLGRLYF	FLU α23	1	
		FLUMP58-66_HC1_24	4-2*01	1-5*01	1*01	CASSQSTGQPQHF	FLU β20	9-2*01	26*01	CAPESGQNFVF	FLU α24	1	
		FLUMP58-66_HC1_25	4-3*01	2-3*01	1*01	CASAILGIRIAGF	FLU β21	22*01	42*01	CAVERGL##GSQGNLIF	FLU α25	1	
		FLUMP58-66_HC1_26	6-5*01	2-7*01	1*01	CPTSNPQTGPNXQNS	FLU β22	17*01	20*01	CATDVINDY*LSF	FLU α26	1	
		FLUMP58-66_HC1_27	9*01	2-3*01	1*01	CASSGFGGTDTQ#F	FLU β23	41*01	49*01	CAAALYTGNQFYF	FLU α27	1	
		FLUMP58-66_HC1_28	10-1*01	1-1*01	1*01	CARGTKQGSN#MNTEAFF	FLU β24	38-1*01	52*01	CAFMDNAGGTSYGKLTF	FLU α28	1	
		FLUMP58-66_HC1_29	10-3*01	2-7*01	1*01	CSPPKDLPYKRNF	FLU β25	2*02	29*01	CAVEDRGEKHXSCL	FLU α29	1	
			19*01	2-7*01	1*01	CASSIRAAYEQYF	**FLU β12**	NA	NA	NA		1	TCRβ CDR3 previously described by: Valkenburg et al., 2016
	HC2											Enriched	
		FLUMP58_HC2_1	19*01	2-1*01	1*01	CASSIMAYNGQFF	FLU β26	12-1*01	56*01	CVGNSE	FLU α30	2	
												Single	
		FLUMP58_HC2_2	19*01	2-7*01	2*01	CASSIRPSYEQYF	**FLU β27**	27*01	42*01	CAGGGSQGNLIF	**FLU α31**	1	TCRα CDR3 previously described by: Gil et al.,2015; Valkenburg et al, 2016; TCRα CDR3 shared with T1D-1; TCRβ CDR3 shared with T1D-1
		FLUMP58_HC2_3	19*01	2-7*01	2*01	CARGGGGSYERYF	FLU β28	27*01	42*01	CAGGGSQGNLIF	**FLU α31**	1	TCRα CDR3 previously described by: Gil et al.,2015; Valkenburg et al, 2016; TCRα CDR3 shared with T1D-1
		FLUMP58_HC2_4	19*01	2-7*01	2*01	CASGGRSSYERYF	FLU β29	27*01	42*01	CAGGGSQGNLIF	**FLU α31**	1	TCRα CDR3 previously described by: Gil et al.,2015; Valkenburg et al, 2016; TCRα CDR3 shared with T1D-1
		FLUMP58_HC2_5	19*01	1-1*01	2*01	CASSIKANTEAFF	FLU β30	23/DV6*01	45*01	CAASSWS*#GLTF	FLU α32	1	
		FLUMP58_HC2_6	19*01	1-2*01	2*01	CASSFFLAAGYPF	FLU β31	41*01	50*01	CAVLKTSYDKVIF	FLU α33	1	
		FLUMP58_HC2_7	19*01	2-1*01	1*01	CASSIGYEQFF	FLU β32	29/DV5*01	42*01	VQQAQ#YGGSQGNLIF	FLU α34	1	
		FLUMP58_HC2_8	19*01	2-1*01	1*01	CASSILG*NEQFF	FLU β33	8-6*02	31*01	CAVSDSQVGDARLMF	FLU α35	1	
		FLUMP58_HC2_9	19*01	2-1*01	2*01	CASSISSSSYNEQFF	FLU β34	17*01	9*01	CATVDYTGGFKTIF	FLU α36	1	
		FLUMP58_HC2_10	19*01	2-3*01	1*01	CASGGLSTDTQ#F	FLU β35	3*01	36*01	CAVRDGTGANNLFF	**FLU α37**	1	TCRα CDR3 previously described by: Naumov et al., 2008; TCRα CDR3 shared with T1D-1
		FLUMP58_HC2_11	19*01	2-3*01	1*01	CASAGIGGSYDTQ#F	FLU β36	13-2*01	3*01	CAEMYSSASKIIF	FLU α38	1	
		FLUMP58_HC2_12	19*01	2-3*01	2*01	CAGGGMGGSYDTQ#F	FLU β37	9-2*01	23*01	CARTYNQGGKLIF	FLU α39	1	
		FLUMP58_HC2_13	19*01	2-3*01	2*02	CASRGRSTDTQ#F	FLU β38	25*01	42*01	CAGNYGGSQGNLIF	FLU α40	1	
		FLUMP58_HC2_14	19*01	2-7*01	2*02	CASSIRRAYERYF	FLU β39	8-6*02	42*01	CAFGGSQGNLIF	FLU α41	1	
		FLUMP58_HC2_15	19*01	2-7*01	2*02	CASRGRSSYEQYF	FLU β40	14/DV4*01	20*01	CAIELQAR##	FLU α42	1	
		FLUMP58_HC2_16	19*01	2-7*01	2*02	CAGSPFGGAYEQYF	FLU β41	25*01	52*01	CAGLVGTSYGKLTF	FLU α43	1	
		FLUMP58_HC2_17	24-1*01	1-2*01	2*01	CAXGGGEYGXXF	FLU β42	3*01	34*01	CAVRAYNTDKLIF	FLU α44	1	
		FLUMP58_HC2_18	25-1*01	2-7*01	1*01	CAXRXXRXEQNF	FLU β43	3*01	34*01	CAVRDGGDKLIF	FLU α45	1	
		FLUMP58_HC2_19	27*01	2-2*01	1*01	CASGPNFNTGELFF	FLU β44	22*01	45*01	CAVEGADGLTF	FLU α46	1	
		FLUMP58_HC2_20	27*01	1-1*01	2*01	CASSLWGGTEAFF	FLU β45	41*01	34*01	CAALNTDKLIF	FLU α47	1	
		FLUMP58_HC2_21	5-4*01	2-5*01	2*01	CASGGGGPETQYF	FLU β46	8-6*02	11*01	CAGSGYSTLTF	FLU α48	1	
		FLUMP58_HC2_22	6-5*01	2-5*01	1*01	CASSRGTRGRSETQYF	FLU β47	8-3*01	44*01	CAVGLNTGTASKLTF	**FLU α49**	1	TCRα CDR3 shared with HC3
		FLUMP58_HC2_23	7-9*01	2-5*01	1*01	CASSDPRGEETQYF	FLU β48	8-6*02	27*01	CAVVYAGKSTF	FLU α50	1	
	HC3											Single	
		FLUMP58_HC3_1	19*01	2-1*01	2*01	CASTGLGP*NEQFF	FLU β49	24*01	49*01	CAFISNTGNQFYF	FLU α51	1	
		FLUMP58_HC3_2	19*02	2-3*01	1*01	CASSTRAADTQYF	**FLU β50**	13-2*01 ,	42*01	CAENSGGGSQGNLIF	FLU α52	1	
		FLUMP58_HC3_3	19*03	2-3*01	1*01	CASSTRAADTQYF	**FLU β50**	8-3*01	44*01	CAVGLNTGTASKLTF	**FLU α49**	1	TCRα CDR3 shared with HC2
		FLUMP58_HC3_4	19*04	2-3*01	1*01	CASSTRAADTQYF	**FLU β50**	38-1*01	42*01	XEXXGGXXRXXX##	FLU α53	1	
		FLUMP58_HC3_5	19*05	1-1*01	2*01	CASRIGXXXGVXF	FLU β51	36/DV7*02	40*01	CAAY#SGTYKYIF	FLU α54	1	
		FLUMP58_HC3_6	19*06	1-5*01	NA	SPPASYTNQPQXG	FLU β52	35*01	33*01	CAGPSDPGDSNYQLIW	FLU α55	1	

Antigen	Donor	TCR name (clonotype)	TRBV	TRBJ	TRBD	TCRβ CDR3 sequence	TCRβ CDR3 name	TRAV	TRAJ	TCRα CDR3 sequence	TCRα CDR3 name	Number cells	Comments
	T1D-1											Enriched	
FluMP58-66		FLUMP58_T1D-1_1	19*01	2-3*01	2*01	CASSGGSKKTQF	FLU β53	29/DV5*01	42*01	CAASAGGGSQGNLIF	**FLU α56**	3	
		FLUMP58_T1D-1_2	19*01	2-7*01	1*01	CASRGRSSYEHYF	FLU β54	26-1*01	53*01	CIVSVLG#SGGSNYKLTF	FLU α57	2	
		FLUMP58_T1D-1_3	19*01	2-1*01	2*01	CASSIRSLDEQFF	FLU β55	25*01	42*01	CAGSGGGSQGNLIF	FLU α58	2	
												Single	
		FLUMP58_T1D-1_4	19*01	2-1*01	2*01	CASSIRSLDEQFF	FLU β55	25*01	42*01	CPVTSLSAYSPNIF	FLU α59	1	
		FLUMP58_T1D-1_5	19*01	2-1*01	1*01	CASSFTKDYSTMF	FLU β56	29/DV5*01	42*01	CTSVQQAL#GGSQGNLIF	**FLU α60**	1	
		FLUMP58_T1D-1_6	19*01	2-3*01	2*01	CAGGRKRNTQYF	FLU β57	29/DV5*01	42*01	CTSVQQAL#GGSQGNLIF	**FLU α60**	1	
		FLUMP58_T1D-1_7	19*01	1-2*01	1*01	CASSRTATHGYTF	FLU β58	3*01	36*01	CAVRDGTGANNLFF	**FLU α37**	1	TCRα CDR3 previously described by: Naumov et al., 2008; TCRα CDR3 shared with HC2
		FLUMP58_T1D-1_8	19*01	2-1*01	2*01	CASSELGGXANGQFF	FLU β59	3*01	37*02	CAVNRHGSSNTGKLIF	FLU α61	1	
		FLUMP58_T1D-1_9	19*01	2-7*01	1*01	CASSDRSSYEQYF	FLU β60	26-1*01	53*01	CIVRQLGTGAVVTIN*#F	FLU α62	1	
		FLUMP58_T1D-1_10	19*01	2-7*01	1*01	CASSTRPSYEQYF	FLU β61	8-6*02	37*02	CAVSSNTGKLIF	FLU α63	1	
		FLUMP58_T1D-1_11	19*01	2-3*01	1*01	CASSGRPTETQYF	FLU β62	25*01	42*01	CAVSYGGSQGNLIF	**FLU α16**	1	TCRα CDR3 shared with HC1;
		FLUMP58_T1D-1_12	19*01	2-1*01	2*01	CAGSVLRGGRNERFF	FLU β63	36/DV7*02	22*01	CAVED#ISSGSARQLTF	FLU α64	1	
		FLUMP58_T1D-1_13	27*01	2-2*01	1*01	CASSLIFPGELFF	FLU β64	8-4*03	45*01	VL*#GGGADGLTF	FLU α65	1	
		FLUMP58_T1D-1_14	27*01	1-5*01	1*01	CASSLMGQGINQPQHF	FLU β65	17*01	23*01	CARSRNKQGGIFFF	FLU α66	1	
		FLUMP58_T1D-1_15	3-2*01	2-3*01	2*01	CASRGA*RGRGVRR#F	FLU β66	8-2*01	52*01	VL*V#AGGTSYGKLTF	FLU α67	1	
		FLUMP58_T1D-1_16	4-3*01	1-4*01	1*01	CASSQEWGINEKLFF	FLU β67	25*01	23*01	CAGSPIYNQGGKLIF	FLU α68	1	
		FLUMP58_T1D-1_17	7-8*01	2-7*01	2*01	CATPTNLGGGGGNYF	FLU β68	29/DV5*01	52*01	VQQDLGY##GGTSYGKLTF	FLU α69	1	
		FLUMP58_T1D-1_18	19*01	2-3*01	2*01	CASSGGSKNYAVF	FLU β69	29/DV5*01	42*01	CAASAGGGSQGNLIF	**FLU α56**	1	
		FLUMP58_T1D-1_19	19*01	1-5*01	1*01	CASSIHGANQPQHF	**FLU β70**	23/DV6*01	33*01	CAASARSMDSNYQLIW	**FLU α70**	1	
		FLUMP58_T1D-1_20	19*01	1-5*01	1*01	CASSIRGANQPQHF	FLU β71	23/DV6*01	33*01	CAASARSMDSNYQLIW	**FLU α70**	1	
		FLUMP58_T1D-1_21	19*01	1-1*01	1*01	CAGSVTGALGTEAFF	FLU β72	17*01	52*01	CATDDHAGGTSYGKLTF	FLU α71	1	
		FLUMP58_T1D-1_22	19*01	2-3*01	1*01	CASRKRGTAPQFF	FLU β73	3*01	42*01	CASPGGGGSQGNLIF	FLU α72	1	
		FLUMP58_T1D-1_23	19*01	2-7*01	1*01	CASRTRSAYEQYF	FLU β74	27*01	42*01	CAGGGSQGNLIF	**FLU α31**	1	TCRα CDR3 previously described by: Gil et al.,2015; Valkenburg et al, 2016; TCRα CDR3 shared with HC2
		FLUMP58_T1D-1_24	19*01	1-5*01	1*01	CASSIHGANQPQHF	**FLU β70**	23/DV6*01	33*01	CAAGGGAWLARISF	FLU α73	1	
		FLUMP58_T1D-1_25	19*01	2-7*01	1*01	CASSIRSAYEQYF	**FLU β75**	29/DV5*01	42*01	CAASATPYGGSQGNLIF	FLU α74	1	
		FLUMP58_T1D-1_26	4-2*01	1-2*01	1*01	CASSPGTPTYGYTF	FLU β76	8-2*01	30*01	CAVSDLIGDDKIIF	FLU α75	1	
		FLUMP58_T1D-1_27	19*01	2-3*01	1*01	CASSPRSTDTQYF	FLU β77	25*01 F	42*01	CAGASGGSQGNLIF	FLU α76	1	TCRα CDR3 previously described by: Gil et al., 2015; Naumov et al., 2008; TCRβ CDR3 previously described by: Lehner et al., 1995; Naumov et al., 1998; Naumov et al., 2008
		FLUMP58_T1D-1_28	18*01 F	2-2*01	1*01	CASSQTGAEELFF	FLU β78	36/DV7*02	32*02	CAVGGATNKLIF	FLU α77	1	
		FLUMP58_T1D-1_29	6-5*01 F	2-5*01	1*01	CASSTLTGAETQYF	FLU β79	24*01	47*01	CAFIHLEYGNKLVF	FLU α78	1	
		FLUMP58_T1D-1_30	27*01	1-1*01	1*01	CASTSRGVSKVFF	FLU β80	14/DV4*02 F	33*01	CAMREVHMDSNYQLIR	FLU α79	1	
		FLUMP58_T1D-1_31	19*01	2-7*01	1*01	CASSIRSSYEQYF	**FLU β81**	26-2*01	42*01	CTPG#SQGNLIF	FLU α80	1	TCRβ CDR3 previously described by: Lehner et al., 1995, Valkenburg et al., 2016
		FLUMP58_T1D-1_32	19*01	2-7*01	1*01	CASSIRSSYEQYF	**FLU β81**	36/DV7*02	45*01	CI##GGADGLTF	FLU α81	1	TCRβ CDR3 previously described by: Lehner et al., 1995, Valkenburg et al., 2016
			NA	NA	NA	NA		23/DV6*01	33*01	CAEKARSMVXNYQLIW	FLU α82	1	
			NA	NA	NA	NA		27*01	27*01	CAGANTNAGKSTF	FLU α83	1	
			NA	NA	NA	NA		22*01	37*02	CAVGPSNTGKLIF	FLU α84	1	
			NA	NA	NA	NA		8-6*01	42*01	CAVSGGNYGVXQGNLIF	FLU α85	1	
			NA	NA	NA	NA		8-4*03	15*01	CCEGLM#AGTALIF	FLU α86	1	
			NA	NA	NA	NA		36/DV7*02	45*01	CI#GGGADGLTF	FLU α87	1	
			NA	NA	NA	NA		26-2*01	10*01	CILRDPFTGGGIKFTF	FLU α88	1	
			NA	NA	NA	NA		26-1*01	2*01	CIVRVLGIVEVATIN#TF	FLU α89	1	
			NA	NA	NA	NA		3*01	8*01	CAVRDLNTGFQKLVF	FLU α90	1	
			NA	NA	NA	NA		26-1*01	52*01	CIVRRGGTSYGKLTF	FLU α91	1	
			NA	NA	NA	NA		29/DV5*01	15*01	VQQYSS#QAGTALIF	FLU α92	1	
			NA	NA	NA	NA		25*01	9*01	CAAEDDTGGFKTIF	FLU α93	1	
			NA	NA	NA	NA		29/DV5*01	42*01	CTSVQQAL#GGSQGNLIF	**FLU α60**	1	
			NA	NA	NA	NA		29/DV5*01	42*01	CTSVQQAL#GGSQGNLIF	**FLU α60**	1	
			NA	NA	NA	NA		23/DV6*01	33*01	CAASARSMDSNYQLIW	**FLU α70**	1	
			NA	NA	NA	NA		3*01	36*01	CAVRDGTGANNLFF	**FLU α37**	1	TCRα CDR3 previously described by: Naumov et al., 2008; TCRα CDR3 shared with HC2
			19*01	1-2*01	2*02	CAGGIGSYGYPF	FLU β82	NA	NA	NA		1	
			19*01	1-2*01	1*01	CASGIGGNGYPF	FLU β83	NA	NA	NA		1	
			19*01	2-7*01	2*01	CASGIRSSYERYF	FLU β84	NA	NA	NA		1	
			19*01	2-5*01	2*02	CASGSIEETQYF	FLU β85	NA	NA	NA		1	
			19*01	2-7*01	1*01	CASGXRXXYEXXF	FLU β86	NA	NA	NA		1	
			19*01	2-7*01	1*01	CASRARSPNEQNF	FLU β87	NA	NA	NA		1	
			19*01	1-1*01	1*01	CASRATGGLGPEAFF	FLU β88	NA	NA	NA		1	
			19*01	1-2*01	2*02	CASRIGINGYPF	FLU β89	NA	NA	NA		1	
			19*01	2-7*01	1*01	CASRIRGGSEXXF	FLU β90	NA	NA	NA		1	
			19*01	2-7*01	1*01	CASRIRGSYEQYF	FLU β91	NA	NA	NA		1	
			19*01	2-5*01	2*02	CASRKRSSEAQYF	FLU β92	NA	NA	NA		1	
			19*01	2-1*01	1*01	CASRSTGDYSTMS#FF	FLU β93	NA	NA	NA		1	
			19*01	1-1*01	1*01	CASSGTXLNTEAFF	FLU β94	NA	NA	NA		1	
			19*01	1-2*01	1*01	CASSIGGNGYPF	FLU β95	NA	NA	NA		1	

Antigen	Donor	TCR name (clonotype)	TRBV	TRBJ	TRBD	TCRβ CDR3 sequence	TCRβ CDR3 name	TRAV	TRAJ	TCRα CDR3 sequence	TCRα CDR3 name	Number cells	Comments
	T1D-1												
FluMP58-66			19*01	2-7*01	1*01	CASSIRGSYEQYF	**FLU β96**	NA	NA	NA		1	
			19*01	2-7*01	2*01	CASSIRPSYERYF	FLU β97	NA	NA	NA		1	
			19*01	2-7*01	2*01	CASSIRSSYERYF	FLU β98	NA	NA	NA		1	
			27*01	1-6*02	1*01	CASSLSLGDRXPYNSPXXF	FLU β99	NA	NA	NA		1	
			28*01	2-7*01	1*01	CASSSWTGXXTXX#F	FLU β100	NA	NA	NA		1	
			19*01	1-2*01	1*01	CASSTGLXGYTF	FLU β101	NA	NA	NA		1	
			19*01	1-2*01	1*01	CASSTGLYGYPF	FLU β102	NA	NA	NA		1	
			7-8*03	2-7*01	1*01	CASSTSLGGGARQYF	FLU β103	NA	NA	NA		1	
			24-1*01	2-7*01	1*01	CATRKFGRKD#EQYF	FLU β104	NA	NA	NA		1	
			29-1*01	2-6*01	1*01	CSVDRQG#SGANVLTF	FLU β105	NA	NA	NA		1	
			19*01	2-7*01	2*01	CVCRXGGSYERDF	FLU β106	NA	NA	NA		1	
			19*01	1-2*01	NA	CVGRKGRDGYPF	FLU β107	NA	NA	NA		1	
			23-1*01	2-7*01	NA	CXRPXXGGGXTLPPTGP	FLU β108	NA	NA	NA		1	
			19*01	2-5*01	NA	CXSSVRSSYEQYF	FLU β109	NA	NA	NA		1	
			19*02	2-7*01	1*01	CXSXIRRPXXQXF	FLU β110	NA	NA	NA		1	
			19*03	1-2*01	NA	CAGXKGSXXPTL	FLU β111	NA	NA	NA		1	
			19*04	1-5*01	2*02	CASGNGNDPPLXF	FLU β112	NA	NA	NA		1	
			19*05	2-7*01	2*01	CASSSRSPYEQYF	FLU β113	NA	NA	NA		1	
			19*06	2-1*01	1*01	CAGRVGSAPAXSC	FLU β114	NA	NA	NA		1	
			27*01	2-7*01	2*01	CAGXFGGGSSKQXF	FLU β115	NA	NA	NA		1	
			23-1*01	2-7*01	NA	CASAKXSGRNSLPXXGX	FLU β116	NA	NA	NA		1	
			5-1*01	2-1*01	1*01	CASCLGQGSYNEQFF	FLU β117	NA	NA	NA		1	
			19*01	1-2*01	1*01	CASGIGGYGYPF	FLU β118	NA	NA	NA		1	
			27*01	2-5*01	1*01	CASSFGQGAETQYF	FLU β119	NA	NA	NA		1	
			19*01	2-7*01	1*01	CASSGRSPYEQDF	FLU β120	NA	NA	NA		1	
			19*01	1-2*01	NA	CASSIGYFGYTF	FLU β121	NA	NA	NA		1	
			19*01	1-5*01	2*02	CASSILSNQPQHF	FLU β122	NA	NA	NA		1	
			19*01	1-5*01	2*01	CASSKRSNPPQHF	FLU β123	NA	NA	NA		1	
			28*01	2-1*01	2*01	CASSLPRDLASGRTKTRSYNEQFF	FLU β124	NA	NA	NA		1	
			28*01	2-7*01	1*01	CASSSWTG#SYEQYF	FLU β125	NA	NA	NA		1	
			28*01	2-7*01	1*01	CASSSWTGGPTSS#F	FLU β126	NA	NA	NA		1	
			19*01	2-5*01	1*01	CASSTRSGETQYF	FLU β127	NA	NA	NA		1	
			19*01	2-7*01	NA	CASSVRSSYEQYF	FLU β128	NA	NA	NA		1	TCRβ CDR3 previously described by: Valkenburg et al. 2016
			19*01	1-2*01	1*01	GASSIGIFGYTF	FLU β129	NA	NA	NA		1	
			19*01	2-7*01	1*01	CASSIRGSYEQYF	**FLU β96**	NA	NA	NA		1	
			19*01	2-7*01	1*01	CASSIRPSYEQYF	**FLU β27**	NA	NA	NA		1	TCRβ CDR3 shared with HC2
			19*01	1-2*01	1*01	CASSTGLGYGYPF	FLU β130	NA	NA	NA		1	
			19*01	1-2*01	2*02	CASRIGIYGYPF	FLU β131	NA	NA	NA		1	
			19*01	2-7*01	1*01	CASSIRSSYEQYF	**FLU β81**	NA	NA	NA		1	TCRβ CDR3 previously described by: Lehner et al., 1995, Valkenburg et al., 2016
			19*01	1-2*01	2*02	CASSTGSYGYTF	FLU β132	NA	NA	NA		1	TCRβ CDR3 previously described by: Lehner et al., 1995; Naumov et al, 1998
			19*01	2-7*01	2*02	CASSIGSYGYTF	FLU β133	NA	NA	NA		1	TCRβ CDR3 previously described by: Naumov et al, 1998, Valkenburg et al. 2016
			19*01	2-1*01	2*01	CASSSRSAIEQFF	FLU β134	NA	NA	NA		1	
			19*02	2-7*02	2*02	CASSIRSAYEQYF	**FLU β75**	NA	NA	NA		1	
			19*01	2-1*01	2*01	CASSIRSLDEQFF	**FLU β55**	NA	NA	NA		1	
			7-2*01	2-7*01	2*01	CASSTSLGGGGRQSF	FLU β135	NA	NA	NA		1	

Antigen	Donor	TCR name (clonotype)	TRBV	TRBJ	TRBD	TCRβ CDR3 sequence	TCRβ CDR3 name	TRAV	TRAJ	TCRα CDR3 sequence	TCRα CDR3 name	Number cells	Comments
	T1D-1											Enriched	
IGRP265-273		IGRP265-273_T1D-1_1	3-1*01	2-4*01	1*01	CASSQDRWDVMSKNIQYF	**IGRP β1**	25*01	53*01	CAGLGDSGGSNYKLTF	**IGRP α1**	299	TCRα CDR3 shared with T1D-3
		IGRP265-273_T1D-1_2	10-3*01	1-2*01	1*01	CAISEMLIRGLANYGYPF	IGRP β2	29/DV5*01	53*01	CAASGGSNYKLTF	**IGRP α2**	20	TCRα CDR3 shared with T1D-2
		IGRP265-273_T1D-1_3	10-3*01	1-2*01	2*02	CAISDRFMREEMTYGYPF	IGRP β3	29/DV5*01	53*01	CAASGGSNYKLTF	**IGRP α2**	11	TCRα CDR3 shared with T1D-2
		IGRP265-273_T1D-1_4	24-1*01	2-1*01	2*01	CATSDPLIKGLAEYNEQFF	IGRP β4	29/DV5*01	53*01	CAASGGSNYKLTF	**IGRP α2**	6	TCRα CDR3 shared with T1D-2
		IGRP265-273_T1D-1_5	6-2*01	2-7*01	1*01	CASGTLLIRGKGYERYF	IGRP β5	29/DV5*01	53*01	CAASGGSNYKLTF	**IGRP α2**	5	TCRα CDR3 shared with T1D-2
		IGRP265-273_T1D-1_6	3-1*01	2-4*01	1*01	CASSQDRWDVMSKNIQYF	**IGRP β1**	17*01	17*01	CATLCPQ#NKLTF	IGRP α3	4	
		IGRP265-273_T1D-1_7	3-1*01	2-4*01	1*01	CASGDGLAGKITGELFF	IGRP β6	25*01	53*01	CAGLGDSGGSNYKLTF	**IGRP α1**	3	TCRα CDR3 shared with T1D-3
		IGRP265-273_T1D-1_8	3-1*01	2-4*01	1*01	CASSQDRWDVMSKNIQYF	**IGRP β1**	29/DV5*01	54*01	CAASLLQGAQKLVF	IGRP α4	2	
		IGRP265-273_T1D-1_9	19*01	1-2*01	2*01	CAKKTSLLGPGANYGYTF	IGRP β7	29/DV5*01	53*01	CAASGGSNYKLTF	**IGRP α2**	2	TCRα CDR3 shared with T1D-2
		IGRP265-273_T1D-1_10	28*01	2-7*01	1*01	CASRGLIIQRSSYEQYF	**IGRP β8**	29/DV5*01	39*01	CAASDGAGNMLTF	IGRP α5	2	
			3-1*01	2-4*01	1*01	CASSQDRWDVMSKNIQYF	**IGRP β1**	17*01	NA	NA		6	
												Single	
		IGRP265-273_T1D-1_11	10-3*01	1-2*01	2*02	CASSGRFLREGMTYGYTF	IGRP β9	29/DV5*02	53*02	CAASGGSNYKLTF	**IGRP α2**	1	TCRα CDR3 shared with T1D-2
		IGRP265-273_T1D-1_12	24-1*01	2-6*01	1*01	CPLRGPLFMGGEGPD#F	IGRP β10	29/DV5*05	53*05	CAASGGSNYKLTF	**IGRP α2**	1	TCRα CDR3 shared with T1D-2
		IGRP265-273_T1D-1_13	3-1*01	2-4*01	1*01	CASSQDRWDVMSQNIQYF	IGRP β11	8-3*01	36*01	CALQTGANNLFF	IGRP α6	1	
		IGRP265-273_T1D-1_14	10-3*01	1-2*01	2*01	CAISDRFMREGMTYGYTF	IGRP β12	29/DV5*01	53*01	VQQAG#SNYKLTF	IGRP α7	1	
		IGRP265-273_T1D-1_15	10-3*01	2-7*01	1*01	CAISES*GYEQYF	IGRP β13	8-3*01	43*01	CAVGEGNDMRF	IGRP α8	1	
		IGRP265-273_T1D-1_16	27*01	2-2*01	2*01	CASSDGLAGK#ANTGELFF	**IGRP β14**	17*01	17*01	CATLC#AGNKLTF	IGRP α9	1	
		IGRP265-273_T1D-1_17	28*01	2-7*01	2*01	CASSSFVIAGGDYEQNF	IGRP β15	29/DV5*01	21*01	CAASVGSNFNKFYF	IGRP α10	1	
		IGRP265-273_T1D-1_18	3-1*01	2-4*01		CASRQDRWEKMGQNIRRF	IGRP β16	25*01	53*01	CAGLGDSGGSNYKLTF	**IGRP α1**	1	TCRα CDR3 shared with T1D-3

Antigen	Donor	TCR name (clonotype)	TRBV	TRBJ	TRBD	TCRβ CDR3 sequence	TCRβ CDR3 name	TRAV	TRAJ	TCRα CDR3 sequence	TCRα CDR3 name	Number cells	Comments
	T1D-1												
IGRP265-273		IGRP265-273_T1D-1_19	6-5*01	2-1*01	2*01	CASSPVPGLDEQFF	IGRP β17	17*01	20*01	CAAFRSNDYKLSF	IGRP α11	1	
		IGRP265-273_T1D-1_20	24-1*01	2-1*01	1*01	CATSDLPYIGTRLNEQFF	IGRP β18	8-4*01	53*01	CAVSADSGGSNYKLTF	IGRP α12	1	
			NA	NA	NA	NA		29/DV5*01	53*01	CAASGGSNYKLTF	**IGRP α2**	1	TCRα CDR3 shared with T1D-2
			NA	NA	NA	NA		25*01	53*01	CAGLGDSGGSNYKLTF	**IGRP α1**	1	TCRα CDR3 shared with T1D-3
			10-3*01	1-2*01	2*02	CAISDRFMRXXXTNGYPF	IGRP β19	NA	NA	NA		1	
			27*01	2-2*01	2*02	CASGHFLAGKIPXGXL#F	IGRP β20	NA	NA	NA		1	
			27*01	2-2*01	2*01	CASSDGLAGKREHRG##FF	IGRP β21	NA	NA	NA		1	
			28*01	2-7*01	1*01	CASRVLIIKKSSYFG*F	IGRP β22	NA	NA	NA		1	
			27*01	2-2*01	2*01	CASSDGLAGK#ANTGELFF	**IGRP β14**	NA	NA	NA		1	
			6-5*01	2-3*01	2*02	CASSYVAPRFGDTQ#F	IGRP β23	NA	NA	NA		1	
			3-1*01	2-4*01	2*01	CAXGXERWXVVSKNIQYF	IGRP β24	NA	NA	NA		1	
			28*01	2-7*01	1*01	CASRGLIIQRSSYEQYF	**IGRP β8**	NA	NA	NA		1	
	T1D-2											**Enriched**	
		IGRP265-273_T1D-2_1	30*01	1-2*01	1*01	CAWGVLFIGRGVGDYGYTF	IGRP β25	29/DV5*01	53*01	CAASGGSNYKLTF	**IGRP α2**	5	TCRα CDR3 shared with T1D-1
		IGRP265-273_T1D-2_2	28*01	2-7	2*01	CASGSHHISGGATXR#	IGRP β26	29/DV5*01	53*01	CAASGGSNYKLTF	**IGRP α2**	2	TCRα CDR3 shared with T1D-1
		IGRP265-273_T1D-2_3	3-1*01	1-2*01	1*01	CASSQEVMTALQPDYGYTF	IGRP β27	29/DV5*01	53*01	CAASGGSNYKLTF	**IGRP α2**	3	TCRα CDR3 shared with T1D-1
			NA	NA	NA	NA		29/DV5*01	53*01	CAASGGSNYKLTF	**IGRP α2**	71	TCRα CDR3 shared with T1D-1
												**Single**	
		IGRP265-273_T1D-2_4	28*01	2-3*01	2*01	CAKSSPXISGITTDXRYF	IGRP β28	29/DV5*01	53*01	CAASGGSNYKLTF	**IGRP α2**	1	TCRα CDR3 shared with T1D-1
		IGRP265-273_T1D-2_5	5-8*01	1-2*01	1*01	CASSSAVIRGLDWCYTF	IGRP β29	29/DV5*01	53*01	CAASGGSNYKLTF	**IGRP α2**	1	TCRα CDR3 shared with T1D-1
		IGRP265-273_T1D-2_6	28*01	2-3*01	2*01	CASSSPFFRGIXXDXGYF	IGRP β30	29/DV5*01	53*01	CAASGGSNYKLTF	**IGRP α2**	1	TCRα CDR3 shared with T1D-1
		IGRP265-273_T1D-2_7	10-3*01	1-2*01	1*01	CAISDLSITGGDNYGYTF	IGRP β31	29/DV5*01	53*01	CAASGGSNYKLTF	**IGRP α2**	1	TCRα CDR3 shared with T1D-1
		IGRP265-273_T1D-2_8	19*01	2-7*01	2*01	CASRSSDPYEQYF	IGRP β32	8-1*01	34*01	CAVISYNTDKLIF	IGRP α13	1	
		IGRP265-273_T1D-2_9	6-1*01	1-2*01	1*01	CASSEVNRGINGYTF	IGRP β33	26-1*01	37*02	CISP*SGSSNTGKLIF	IGRP α14	1	
		IGRP265-273_T1D-2_10	6-1*01	1-2*01	2*02	CASSPRXKANYGYTF	IGRP β34	3*01	26*01	CAVRDFSDNYGQNFVF	IGRP α15	1	
		IGRP265-273_T1D-2_11	4-2*01	2-2*01	2*02	CASSQGVIESGELFF	IGRP β35	26-1*01	39*01	CIXXL*NDIGENMFLI	IGRP α16	1	
		IGRP265-273_T1D-2_12	5-8*01	1-2*01	1*01	CASSSAVIRGLDGGYTF	IGRP β36	26-1*01	29*01	CIVRVA#SGNTPLVF	IGRP α17	1	
			27*01	1-6*02	1*01	CASNTGLRFTPP#F	IGRP β37	NA	NA	NA		1	
			2*01	1-5*01	1*01	CASSEGAAIGPQHF	IGRP β38	NA	NA	NA		1	
			7-8*01	2-7*01	1*01	CASSLGDNGGYEQYF	IGRP β39	NA	NA	NA		1	
			28*01	1-5*01	2*01	CASSLSFSXGGGXQPQHF	IGRP β40	NA	NA	NA		1	
			12-3*01	1-2*01	1*01	CASSLWVAGNGSTF	IGRP β41	NA	NA	NA		1	
			4-2*01	2-2*01	2*02	CASSQDPGXGELFF	IGRP β42	NA	NA	NA		1	
			28*01	2-7*01	2*01	CASSSHHISGGXTSR#F	IGRP β43	NA	NA	NA		1	
			28*01	2-3*01	1*01	CASSSPHFIEIXTDTQXF	IGRP β44	NA	NA	NA		1	
	T1D-3											**Enriched**	
		IGRP265-273_T1D-3_1	3-1*01	2-2*01	1*01	CASSQDHRVMRLDAGELFF	**IGRP β45**	25*01	53*01	CAGLGDSGGSNYKLTF	**IGRP α1**	20	TCRα CDR3 shared with T1D-1
			NA	NA	NA	NA		26-1*03	54*01	CIAQGAQKLVF	**IGRP α18**	7	
			3-1*01	2-2*01	1*01	CASSQDHRVMRLDAGELFF	**IGRP β45**	NA	NA	NA		23	
												**Single**	
		IGRP265-273_DM3_2	6-1*01	2-7*01	1*01	CAIWKRV#YEQYF	IGRP β46	8-6*02	13*02	CAVTRW#GGYQKVTF	IGRP α19	1	
		IGRP265-273_DM3_3	7-8*01	2-5*01	1*01	CASRIWYIRNEETQYF	IGRP β47	25*01	47*02	CAG#YGNKLVF	IGRP α20	1	
		IGRP265-273_DM3_4	29-1*01	1-2*01	1*01	CSVPTEGIYGYTF	IGRP β48	35*01	28*01	CAXSGGGSYQLTF	IGRP α21	1	
		IGRP265-273_DM3_5	5-6*01	2-7*01	1*01	CASSLDRAIGVTYEQYF	IGRP β49	8-2*01	32*02	CVVSDGYGGATNKLIF	IGRP α22	1	
		IGRP265-273_DM3_6	5-6*01	2-1*01	2*01	CASSYGLAEQFF	IGRP β50	8-6*02	21*01	CALPP#YNFNKFYF	IGRP α23	1	
		IGRP265-273_DM3_7	20-1*01	2-6*01	1*01	CSASASLTSGANVLTF	IGRP β51	26-1*01	54*01	CIAQGAQKLVF	**IGRP α18**	1	
		IGRP265-273_DM3_8	6-1*01	2-1*01	2*01	CARRSRGENNGGVF	IGRP β52	27*01	23*01	CAGGLIYNQEGKLIF	IGRP α24	1	
		IGRP265-273_DM3_9	6-6*01	2-7*01	NA	CASSYQHEQYF	IGRP β53	8-3*01	45*01	CAGRLVVGGGADALTF	IGRP α25	1	
			NA	NA	NA	NA		4*01	42*01	CLAGEWYGGSQGNLIF	IGRP α26	1	
			NA	NA	NA	NA		26-1*01	39*01	CIVRVAMGAGNMLTF	IGRP α27	1	
			NA	NA	NA	NA		17*01	45*01	CATDGAGGADGLTF	IGRP α28	1	
			NA	NA	NA	NA		8-4*05	39*01	CVVSLNNAGNMLTF	IGRP α29	1	
			2*01	1-5*01	2*01	CASSDGGGGQPXHF	IGRP β54	NA	NA	NA		1	
			6-1*01	2-2*01	1*01	CASSEDRGDTGELFF	IGRP β55	NA	NA	NA		1	
			6-2*01	2-1*01	1*01	CASSLGRINEQFF	IGRP β56	NA	NA	NA		1	
			4-1*01	2-7*01	1*01	CAXGKKGTPX##F	IGRP β57	NA	NA	NA		1	
			19*01	1-2*01	2*01	FLGARMXGKMLTINTP#	IGRP β58	NA	NA	NA		1	
			6-5*01	1-1*01	1*01	CAIREDSVFFF	IGRP β59	NA	NA	NA		1	
			28*01	1-4*01	1*01	CASRPTTGLKLFF	IGRP β60	NA	NA	NA		1	
			3-1*01	2-3*01	1*01	CASSHGKTQXF	IGRP β61	NA	NA	NA		1	
			12-3*01	2-2*01	2*01	CASSLGGGGLFF	IGRP β62	NA	NA	NA		1	
			4-2*01	2-6*01	1*01	CASSQRQGSGANVLTF	IGRP β63	NA	NA	NA		1	
			20-1*01	2-7*01	1*01	CSARGXSSYEQYF	IGRP β64	NA	NA	NA		1	
			27*01	2-3*01	2*01	CARSRGGXTKTQ#F	IGRP β65	NA	NA	NA		1	
			4-2*01	2-7*01	2*01	CASXPREXSSXERXF	IGRP β66	NA	NA	NA		1	
	T1D-4											**Single**	
		IGRP265-273_T1D-4_1	6-5*01	2-2*01	1*01	CASVGPGYTGELFF	IGRP β67	8-2*01	48*01	CAVS#SNFGNEKLTF	IGRP α30	1	
		IGRP265-273_T1D-4_2	27*01	2-4*01	1*01	CASREGSYPKNIQYF	IGRP β68	8-6*01	29*01	CKAPLVF	IGRP α31	1	
			NA	NA	NA	NA		3*01	31*01	CAVRLGGARLMF	IGRP α32	1	
			NA	NA	NA	NA		2*01	12*01	VL*#DSSYKLIF	IGRP α33	1	

Antigen	Donor	TCR name (clonotype)	TRBV	TRBJ	TRBD	TCRβ CDR3 sequence	TCRβ CDR3 name	TRAV	TRAJ	TCRα CDR3 sequence	TCRα CDR3 name	Number cells	Comments
	**T1D-4**												
IGRP265-273			4-1*01	2-3*01	1*01	CARXGAXXRXE*PXX	IGRP β69	NA	NA	NA		1	
			4-1*01	1-1*01	1*01	CASPRFGEXAR*LLS#FF	IGRP β70	NA	NA	NA		1	
			6-1*01	1-6*02	2*01	CASRGGGSPLHF	IGRP β71	NA	NA	NA		1	
			5-5*01	1-2*01	1*01	CASSLTGTASTYGYTF	IGRP β72	NA	NA	NA		1	
			27*01	1-1*01	2*02	VPPVCV*GGTLKL#F	IGRP β73	NA	NA	NA		1	
	**T1D-5**											**Single**	
		IGRP265-273_T1D-5_1	12-3*01	1-1*01	1*01	CASSWETETEAFF	IGRP β74	8-1*01	9*01	CAVMAPHTGGFKTIF	IGRP α34	1	
		IGRP265-273_T1D-5_2	6-5*01	2-3*01	2*01	CASSYSGGNRKNTQNF	IGRP β75	29/DV5*01	52*01	CAASASGGTSYGKLTF	IGRP α35	1	
		IGRP265-273_T1D-5_3	12-3*01	1-2*01	2*01	FASRLGGSYX*TF	IGRP β76	36/DV7*02	42*01	CAAMNYGGSQGNLIF	IGRP α36	1	
			NA	NA	NA	NA		8-1*01	43*01	CAVLMDNDMRF	IGRP α37	1	
			NA	NA	NA	NA		8-2*01	33*01	CAVTRNPGDSNYQLIW	IGRP α38	1	
			6-2*01	2-1*01	2*01	CASCSTSFNEQFF	IGRP β77	NA	NA	NA		1	
			2*01	1-2*01	2*01	CASSDSIG*LWL##F	IGRP β78	NA	NA	NA		1	
			6-2*01	2-7*01	2*01	CASSNRXSFYERYF	IGRP β79	NA	NA	NA		1	
	**T1D-12**											**Single**	
			NA	NA	NA	NA		3*01	22*01	CADS#ARQLTF	IGRP α39	1	
			NA	NA	NA	NA		8-4*04	17*01	CAVSEIKAAGNKLTF	IGRP α40	1	
			NA	NA	NA	NA		41*01	42*01	CAVAAGYGGSQGNLIF	IGRP α41	1	
			7-9*01	2-3*01	1*01	CASSLILGAPDTX#F	IGRP β80	NA	NA	NA		1	
	**T1D-13**											**Single**	
			29-1*01	2-3*01	2*01 F	CSPTGLRTDTQYF	IGRP β81	NA	NA	NA		1	
			29-1*01	2-7*01	1*01 F	CSVESMDRNYEQYF	IGRP β82	NA	NA	NA		1	
	**T1D RO-3**											**Single**	
		IGRP265-273_ROTDM1_1	28*01	2-1*01	2*01	CASSPITGTSLANEQFF	IGRP β83	12-1*01	11*01	CVVKRGYSTLTF	IGRP α42	1	
		IGRP265-273_ROTDM1_2	6-2*01	2-7*01	2*01	FXGSPGTGGGKXYF	IGRP β84	6*03	11*01	CALRGGYSTLTF	IGRP α43	1	
			NA	NA	NA	NA		25*01	15*01	CAGPVHQAGTALIF	IGRP α44	1	
			NA	NA	NA	NA		12-1*01	31*01	CVGGNARLMF	IGRP α45	1	
			10-2*01	2-7*01	1*01	CAISQQSSYKQYF	IGRP β85	NA	NA	NA		1	
	**HC5**											**Single**	
		IGRP265-273_HC5_1	10-3*01	1-1*01	2*02	CAIRGEGNTEAFF	IGRP β86	29/DV5*01	40*01	CAASGXGTYKYIF	IGRP α46	1	
			NA	NA	NA	NA		3*01	34*01	CAVRAYLSYNTDKLIF	IGRP α47	1	
			2*01	1-2*01	2*02	CASPGGGTYYGYPF	IGRP β87	NA	NA	NA		1	
			6-1*01	2-4*01		CAXXGXRAVDIQXF	IGRP β88	NA	NA	NA		1	
	**HC8**											**Single**	
		IGRP265-273_HC8_1	10-3*01	2-7*01	2*01	CAISDWSTYEQYF	IGRP β89	29/DV5*01	34*01	CAARVENNTAKLIF	IGRP α48	1	
		IGRP265-273_HC8_2	28*01	2-1*01	1*01	CASSLVGGXXRRSYTGQVF	IGRP β90	6*05	45*01	CALGGYSGGGADGLTF	IGRP α49	1	
			NA	NA	NA	NA		29/DV5*01	48*01	CAASDNFGNEKLTF	IGRP α50	1	
			NA	NA	NA	NA		25*01	48*01	CAGPRSNFGNEKLTF	IGRP α51	1	
			2*01	1-2*01	NA	CASRXSANYGYTF	IGRP β91	NA	NA	NA		1	
			27*01	2-7*01	1*01	CASSLSWGYEQYF	IGRP β92	NA	NA	NA		1	
			14*01	1-1*01	NA	CASSPSRQSLNTEAFF	IGRP β93	NA	NA	NA		1	
			10-3*01	2-7*01	1*01	CATSGTGYYEQYF	IGRP β94	NA	NA	NA		1	
	**HC9**											**Single**	
			NA	NA	NA	NA		29/DV5*01	53*01	CAASGSSNYHLSF	IGRP α52	1	
			NA	NA	NA	NA		29/DV5*01	29*01	CAASPPSGNTPLVF	IGRP α53	1	
			3-1*01	2-7*01	NA	CASRPLLETYDQPL	IGRP β95	NA	NA	NA		1	
	**HC10**											**Single**	
			NA	NA	NA	NA		29/DV5*01	13*02	VILGV#QKVTF	IGRP α54	1	
			NA	NA	NA	NA		25*01	37*02	CAGTPSNTGKLIF	IGRP α55	1	
			6-1*01	2-1*01		CASSEFNEQFF	IGRP β96	NA	NA	NA		1	

Supplementary Table S2: Overview of samples used in the study.

Sample group	N	Sex (m/f)	Median age, y (range)	Median time after onset, y (range)	Figure(s) with data
Frequency and single cell TCR					
TCR analysis (Flu)	4	4/-	54 (31.2-63.0)		Fig. S1
Recent onset type 1 diabetes*	6	4/2	9.5 (7.4-10.2)	0.02 (0.01-0.04)	Fig.1
Healthy control children	8	4/4	10.5 (7.3-10.8)		Fig.1
Type 1 diabetes (>1 y duration)	13	4/9	34.4 (23.3-45.9)	14.3 (1.1-41.5)	Fig.1, Fig S2
Healthy (adult)	10	4/6	47.4 (24.0-60.0)		Fig. S2
TCRα NGS					
Healthy control adolescents	14	4/10	13.9 (11.7-16.5)		Fig.3, Fig. S5
Islet autoantibody positive	13	10/3	11.6 (3.9-18.7)		Fig.3, Fig. S5
Recent onset type 1 diabetes*	8	3/5	19.4 (5.3-22.9)	0.4 (0.04-0.93)	Fig.3, Fig. S5

*No overlap between patients

Supplementary Table S3: TCR gene usage and CDR3 sequence information of CD8$^+$ T cell clones

Antigen	Clone name	TRBV	TRBJ	TRBD	TCRβ CDR3 sequence	TRAV	TRAJ	TCRα CDR3 sequence
$\text{FluMP}_{58\text{-}66}$	clone 7	6-1*01	1-5*01	1*01	CASSDFQAINQPQHF	27*01	23*01	CAGGLTYNQGGKLIF
$\text{IGRP}_{265\text{-}273}$	clone 16 and clone 17	3-1*01	2-4*01	1*01	CASSQDRWDVMSKNIQYF	25*01	53*01	CAGLGDSGGSNYKLTF
$\text{IGRP}_{265\text{-}273}$	clone 22 and clone 27	10-3*01	1-2*01	2*02	CAISDRFMREGMTYGYTF	29/DV5*01	53*01	CAASGGSNYKLTF

Supplementary Methods

Gene expression profiling

Gene expression profiles on single cells were examined as previously described[6] with some modifications. cDNA was synthesized with Quanta qScript TM cDNA Supermix directly on cells. Total cDNA was pre-amplified for 16 cycles (1x 95ºC 8', 95ºC 45'', 49ºC with 0.3ºC increment/cycle 1', 72ºC 1.5') and 1x 72ºC 7' with the TATAA GrandMaster Mix (TATAA Biocenter, Göteborg, Sweden) in a final volume of 35 µl in the presence of primer pairs for the following genes: *CD52, CCL4, FASLG, IL7R, IFNG, CD3E, E2F1, PDCD1, TNF, PRF1, CCR7, GATA3, CCL5, IL10, IL21, GZMA, REL, EOMES, IL17F, GZMB, RGS16, TBX21, GZMH, TNFSF10, LAG3* (25nM final for each primer, see list below for primer details). Pre-amplified cDNA (10 µl) was then treated with 1.2 U Exonuclease I and expression quantified by real time PCR on the BioMark™ HD System (© Fluidigm Corporation, CA, USA) using the 96.96 Dynamic Array IFC and the GE 96x96 Fast PCR+ Melt protocol and SsoFast EvaGreen Supermix with Low ROX (BIO RAD, CA, USA) with 5 µM primers for each assay. Raw data was analyzed using the Fluidigm Real-Time PCR analysis software. Pre-processing and data analysis was conducted using KNIME 2.11.2 and RStudio Version 0.99.486 (Boston, MA, USA). Pre-processing via a linear model to correct for confounding sampling effects was conducted as previously described[6]. To model the bi-modal gene expression of single cells from T cell clones, the Hurdle model, a semi-continuous modeling framework, was applied to the pre-processed data[7]. This allowed us to assess the differential expression profiles with respect to the frequency of expression and the positive expression mean via a likelihood ratio test.

Primers used for gene expression profiling

Gene	Primer preamplification 5'	Primer preamplification 3'	Primer qPCR 5'	Primer qPCR 3'
GZMH	CAGCCATTCCTC CTCCTGT	GAGCAGCTGTCA GCACAAAG	TCCTCCTGTTGGC CTTTCTT	GAGCAGCTGTCAG CACAAAG
TBX21	CCGTGACTGCCT ACCAGAAT	ATCTCCCCCAAG GAATTGAC	CCGTGACTGCCTA CCAGAAT	ATCTCCCCCAAGG AATTGAC
CD52	GCGCTTCCTCTT CCTCCTAC	CTGAAGCAGAAG AGGTGGATT	GCGCTTCCTCTTC CTCCTAC	CTGAAGCAGAAGA GGTGGATT
TNF	CCCCAGGGACCT CTCTCTAA	TGAGGTACAGGC CCTCTGAT	CCCGAGTGACAAG CCTGTAG	TGAGGTACAGGCC CTCTGAT
IL7R	GGAGCCAATGAC TTTGTGGT	CTGCAGGAGTGT CAGCTTTG	GGAGCCAATGACT TTGTGGT	CTGCAGGAGTGTC AGCTTTG
CCL4	CTGTCCTGTCTCT CCTCATGC	GCTTGCTTCTTTT GGTTTGG	TAGCTGCCTTCTG CTCTCCA	GCTTGCTTCTTTTG GTTTGG
EOMES	CACAAATACCAA CCCCGACT	GGGACAATCTGA TGGGATGA	CACAAATACCAAC CCCGACT	GGGACAATCTGAT GGGATGA
TNFSF10	GACAGACCTGCG TGCTGAT	CAGCAGGGGCTG TTCATACT	CCTGCAGTCTCTC TGTGTGG	CAGCAGGGGCTGT TCATACT
CD3E	GCACTCACTGGA GAGTTCTGG	CCTCATCACCGC CTATGTTT	GCACTCACTGGAG AGTTCTGG	CCTCATCACCGCC TATGTTT
E2F1	GCCATCCAGGAA AAGGTGT	TCTGCACCTTCA GCACCTC	GCCATCCAGGAAA AGGTGT	TCTGCACCTTCAG CACCTC
GZMA	TGCAAAAGACTG GGTGTTGA	TTTTTGCTTTTTC CATCAGC	TGCAAAAGACTGG GTGTTGA	TTTTTGCTTTTTCC ATCAGC
GZMB	GGTGGCTTCCTG ATACGAGA	GCTGCAGTAGCA TGATGTCG	ACTGTTGGGGAAG CTCCATA	GCTGCAGTAGCAT GATGTCG
IL21	TCGCCACATGAT TAGAATGC	AAGCAGGAAAAA GCTGACCA	TCGCCACATGATT AGAATGC	AAGCAGGAAAAAG CTGACCA
IFNG	CTGTTACTGCCA GGACCCAT	TGGATGCTCTGG TCATCTTT	GGTCATTCAGATG TAGCGGA	TGGATGCTCTGGT CATCTTT
LAG3	ATCACCACTTAG CGGAAAGC	CTTGGCAGTGAG GAAAGACC	ATCACCACTTAGC GGAAAGC	CTTGGCAGTGAGG AAAGACC
PDCD1	GCTTCCGTGTCA CACAACTG	GCACTTCTGCCC TTCTCTCT	GCTTCCGTGTCAC ACAACTG	GCACTTCTGCCCT TCTCTCT
PRF1	AACTTTGCAGCC CAGAAGAC	GGGTGCCGTAGT TGGAGATA	ACAGCTTCAGCAC TGACACG	GGGTGCCGTAGTT GGAGATA
CCL5	CGCTGTCATCCT CATTGCTA	ACACACTTGGCG GTTCTTTC	ATCTGCCTCCCCA TATTCCT	ACACACTTGGCGG TTCTTTC
REL	ACAAATGTGAAG GGCGATCA	CCGTCTCTGCAG TCTTTTCC	GGAGCACAGCACA GACAACA	CCGTCTCTGCAGT CTTTTCC
RGS16	CACGCTTTCCTG AAGACAGA	GACCTCTTTAGG GGCCTCAC	CACGCTTTCCTGA AGACAGA	GACCTCTTTAGGG GCCTCAC
FASLG	GGGATGTTTCAG CTCTTCCA	CAGAGGCATGGA CCTTGAGT	CAGAAGGAGCTG GCAGAACT	CAGAGGCATGGAC CTTGAGT
IL17F	TCCAAAAGCCTG AGAGTTGC	ATGCAGCCCAAG TTCCTACA	GCCTGTGCCAGGA GGTAGTA	ATGCAGCCCAAGT TCCTACA
IL10	TGCTGGAGGACT TTAAGGGTTA	GCCTTGCTCTTG TTTTCACAG	TTTAAGGGTTACC TGGGTTGC	GCCTTGCTCTTGTT TTCACAG
GATA3	CCGCCCTACTAC GGAAACTC	TTGGAGAAGGGG CTGAGAT	CCGCCCTACTACG GAAACTC	TTGGAGAAGGGGC TGAGAT
**CCR7**	CAATGAAAAGCG TGCTGGT	ATAGGGAGGAAC CAGGCTTT	GTGGTGGCTCTCC TTGTCAT	ATAGGGAGGAACC AGGCTTT

References

1 Valkenburg, S. A. *et al.* Molecular basis for universal HLA-A*0201-restricted CD8+ T-cell immunity against influenza viruses. *Proceedings of the National Academy of Sciences of the United States of America* **113**, 4440-4445, doi:10.1073/pnas.1603106113 (2016).

2 Gil, A., Yassai, M. B., Naumov, Y. N. & Selin, L. K. Narrowing of human influenza A virus-specific T cell receptor alpha and beta repertoires with increasing age. *Journal of virology* **89**, 4102-4116, doi:10.1128/JVI.03020-14 (2015).

3 Naumov, Y. N. *et al.* Multiple glycines in TCR alpha-chains determine clonally diverse nature of human T cell memory to influenza A virus. *J Immunol* **181**, 7407-7419 (2008).

4 Lehner, P. J. *et al.* Human HLA-A0201-restricted cytotoxic T lymphocyte recognition of influenza A is dominated by T cells bearing the V beta 17 gene segment. *The Journal of experimental medicine* **181**, 79-91 (1995).

5 Naumov, Y. N., Hogan, K. T., Naumova, E. N., Pagel, J. T. & Gorski, J. A class I MHC-restricted recall response to a viral peptide is highly polyclonal despite stringent CDR3 selection: implications for establishing memory T cell repertoires in "real-world" conditions. *J Immunol* **160**, 2842-2852 (1998).

6 Bonifacio, E. *et al.* Effects of high-dose oral insulin on immune responses in children at high risk for type 1 diabetes: the Pre-POINT randomized clinical trial. *Jama* **313**, 1541-1549, doi:10.1001/jama.2015.2928 (2015).

7 McDavid, A. *et al.* Modeling bi-modality improves characterization of cell cycle on gene expression in single cells. *PLoS computational biology* **10**, e1003696, doi:10.1371/journal.pcbi.1003696 (2014).
